# Being Female Matters: Drivers of Spatial Genetic Structure in Clonal White Poplar

**DOI:** 10.3390/ijms27208955

**Published:** 2026-10-09

**Authors:** Magdalena Terlecka, Grzegorz Iszkuło, Monika Dering, Mariola Rabska, Łukasz Walas, Katarzyna Sękiewicz

**Affiliations:** 1 Institute of Dendrology, Polish Academy of Sciences, 62-035 Kórnik, Poland; mterlecka@man.poznan.pl (M.T.); iszkulo@man.poznan.pl (G.I.); mrabska@man.poznan.pl (M.R.); lukaswalas@man.poznan.pl (Ł.W.); 2 Institute of Biological Sciences, University of Zielona Góra, 65-417 Zielona Góra, Poland; 3 Faculty of Forestry and Wood Technology, Poznań University of Life Sciences, 60-637 Poznań, Poland; monika.dering@up.poznan.pl

**Keywords:** clonal growth, dioecy, management of genetic resources, population genetics, riparian forests, sex-specific spatial genetic structure

## Abstract

In plants, Spatial Genetic Structure (SGS) is shaped by gene dispersal and effective population density and is further modulated by life-history traits such as clonality and dioecy. However, the combined effects of these factors on SGS remain unclear, particularly in tree species. Here, we examined how clonal reproduction shapes SGS and whether dioecy compounds this pattern in *Populus alba* (white poplar)*,* a key component of riparian forests. Using 15 nuclear microsatellite markers and population genetic analyses of 1,068 individuals, we investigated sex-specific patterns in semi-natural and urban stands. In the studied stands, clonal growth predominated, with compact clonal architecture and occasional long-distance spread. Our results indicate that clonality intensifies SGS, with a nearly twofold stronger effect at the ramet level than at the genet level. Our results support the assumption that differences in clonal architecture between female and male individuals can generate sex-specific spatial patterns and thereby influence population genetic structure, with stronger SGS in female ramets (*Sp* = 0.129 and 0.177 in the semi-natural and urban stands, respectively) than in male ramets (*Sp* = 0.115 and 0.066). A comparison of semi-natural and urban stands indicates that the intensity of spatial genetic structure reflects the combined effects of clonal structure and habitat-specific factors. Spatio-temporal variation in resource availability and disturbance regimes may contribute to these patterns by influencing population dynamics. Given the degradation of Europe’s riparian forests and the limited knowledge of genetic resources of white poplar, understanding these spatial processes is crucial for informing restoration efforts and the sustainable management of genetic resources of the species. Our study revealed that the observed SGS patterns are strongly habitat-dependent; therefore, the protection and restoration of dynamic riparian forests should adopt an integrated genetic–environmental approach to ensure their long-term adaptive potential under ongoing climate change.

## 1. Introduction

Unravelling the genetic and demographic processes that influence Spatial Genetic Structure (SGS), and their interactions, is crucial for understanding the ecological and evolutionary dynamics of plant populations [1,2]. The non-random spatial distribution of genotypes within populations, which refers to SGS, is primarily determined by genetic drift, spatially restricted gene flow, and selection [3,4]. The extent and pattern of SGS can be further modulated by combinations of species-specific life-history traits, such as life-form, reproductive and growth strategies, ecological and demographic traits, including age, sex structure, population density, and reproductive success, which interact with environmental heterogeneity and habitat disturbance [3,5,6].

It has been demonstrated that clonal reproduction can modulate the intensity of SGS in plant populations [7,8,9], including woody species [8,9,10,11]. Recently, growing attention has also been given to the role of dioecy in shaping SGS patterns [9,12,13,14]. Nevertheless, the compound effect of clonality and dioecy on SGS in trees remains poorly understood [2]. Investigating factors influencing SGS in tree populations is therefore essential for understanding how clonality and dioecy can modify the rate and outcomes of microevolutionary processes in this very unique group of plants. This knowledge is a baseline for informing long-term predictions about the demographic and genetic viability of trees, which is especially relevant in terms of the impact of anthropogenic disturbances and habitat fragmentation on riparian forests [15,16].

Substantial SGS may be compounded in clonal plant species [4]. This is because clonal recruitment affects the spatial distribution of genotypes in a population, potentially mimicking reduced intra-population gene flow and resulting in significant SGS if the clonal offspring are recruited at close distances [17,18]. Furthermore, clonality affects mating opportunities substantially [19,20], potentially resulting in increased inbreeding and relatedness, which also enhance SGS [5,21,22]. Some studies demonstrate that the clonal growth strategy has a particularly significant impact on the development of SGS [4,5,23]. For example, strong SGS is observed in the *phalanx* growth type, which manifests with short internodes between ramets and limited mixing of different genotypes due to the aggregation of ramets of a single clone [4,14]. In contrast, the correlation between genetic and spatial distances tends to be weaker in species with *guerrilla* clonal growth, where ramets are more widely spaced, and genets often intermingle. The *phalanx* growth pattern has been reported in many tree species [3].

Dioecy appears to be more prevalent in riparian ecosystems, especially among tree species from the genera *Populus* and *Salix* [24]. This fact may highlight the importance of dioecy-related adaptations (e.g., sex-dependent tolerance to stress, resource acquisition or demands) for riparian species to cope with resource zonation and disturbances, typical for these communities [25]. Generally, it has been shown that dioecy tends to increase the distances between male and female individuals compared to monoecious species, which would weaken the intensity of SGS [26]. Nonetheless, studies have demonstrated that dioecy promotes stronger SGS in dioecious species compared to monoecious species [9,12]. Chybicki et al. [13] demonstrated that deviations from a balanced sex ratio can reduce effective population size and thereby enhance SGS. Also, stronger SGS among dioecious species may reflect the effect of the seed-shadow handicap, as in dioecious plants, the seeds are dispersed exclusively by females, which reduces overall dispersal, affecting the development of SGS [9,27]. In dioecious and clonal species, sex-related differences can also be revealed in terms of clonal traits such as clonal architecture, genet size or number of ramets [9,28,29]. It has often been assumed that females invest more energy in fruit and seed production, while males, due to the lower cost of reproduction, may invest resources in clonal growth [18,30]. In males, clonality may not only compensate for lower reproductive costs but also actively enhance male reproductive success by expanding pollen dispersal potential [31]. Consequently, sex-dependent clonal traits may result in specific demographic and genetic patterns [32,33] that influence the development of various SGS patterns.

The impact of clonal reproduction on SGS in a sex-specific context has not been thoroughly investigated, especially in long-lived clonal trees. Dering et al. [9] have provided evidence that sex-specific clonal traits as well as the spatial segregation of sexes may jointly contribute to the divergent patterns of SGS between the sexes in *Populus alba* (white poplar). However, given that the study was based on a single population, it is difficult to draw general conclusions about how clonality and dioecy shape SGS in tree species. Here, we investigate this issue further by examining the sex-specific patterns of SGS in two white poplar populations located in the divergent habitat contexts: urban and semi-natural, with different levels of anthropogenic and ecological disturbance.

White poplar is a dioecious, clonal tree associated with river valleys and floodplains across central and southern Europe, northern Africa and central Asia [34]. The species is a component of ecologically diverse and productive floodplain forests, which are the most threatened forest ecosystems worldwide, with losses exceeding 50% due to anthropogenic pressure, such as hydrological alterations and land use changes [35]. In Europe, the decline is even more severe, with an estimated 80–90% reduction in floodplain forests, which makes them a conservation and management priority habitat [35,36]. Remaining floodplain communities are highly fragmented and often degraded [37], yet they play a crucial role in carbon cycling and provide essential ecosystem services and benefits to societies, which make them a target of conservation and restoration programmes [38,39,40]. However, management and restoration of clonal plants is challenging [41], as the extent of clonality must be known to design appropriate sampling strategies, to avoid sampling the same clones, and to maintain genetic diversity and, thus, adaptive potential.

Previous studies have reported on the clonal and genetic structure of white poplar at regional or local scales, primarily in the context of phylogeography or hybridisation with European aspen (*Populus tremula*) [11,23,42,43,44,45,46,47]. However, detailed insights into the spatial patterns of genetic variation within populations of this species in relation to clonal structure remain scarce [9]. Floodplain forests face substantial risks from local habitat modifications, such as river regulation and alterations in hydrological patterns, that regulate the survival and recruitment of trees, which are the founding species of these communities [48]. Furthermore, given that they frequently display sex-dependent adaptations or specialisations, dioecious species are predicted to be highly vulnerable to climate change [49]. This is because one sex may be more susceptible to the disturbances imposed by climate change, resulting in an imbalanced sex ratio posing a threat to population survival. Therefore, recognising the SGS patterns in terms of clonal structure and dioecy is indispensable for white poplar populations as it provides insights into the direction of demo-genetic processes. This knowledge is essential for efficient management and conservation of white poplar genetic resources. The species has recently been recognised as important for genetic conservation in riparian ecosystems [50].

By applying nuclear microsatellite markers (nSSRs) with spatially explicit population genetics analyses, we aim to contribute to the understanding of the processes driving spatial genetic structure (SGS) in clonal dioecious white poplar. We address the following questions: 1) Does clonality contribute significantly to SGS in the studied populations? If yes, we expect to find stronger SGS at the ramet level compared to the genet level. 2) Do female and male individuals differ in clonal structure? If yes, could the aggregation of ramets in male and female genets, combined with spatial segregation of sexes, potentially enhance and differentiate SGS between sexes? 3) Is habitat context a significant driver of SGS and clonal patterns in white poplar populations? If yes, we would expect weaker SGS in the semi-natural landscape compared to the urban site, and different clonal architecture in both populations. We discuss our results in the context of increasing attention being paid to understanding the ecological processes that shape the genetic diversity of riparian tree species facing severe habitat degradation [50].

## 2. Results

### 2.1. Genetic Diversity

Linkage disequilibrium was detected between two pairs of loci (ORPM21 and ORPM312-2; ORPM21 and WPMS6) in both populations. Excluding ORPM21 from analyses showed no significant impact; genetic diversity, clonal diversity, and SGS analyses were unchanged, and therefore we left this locus in downstream analyses (Figure S1). Details of the polymorphism observed at the SSRs are provided in Table S1 (Supplementary File). Rarefaction curves for the expected number of multilocus genotypes (eMLG) approached saturation, suggesting that most multilocus genotypic diversity within the study sites was captured (Figure S2, Supplementary File).

All 1068 sampled individuals were successfully genotyped. At the population level, 46 alleles were detected in the RLP and 42 alleles in the DF, with an average of 2.7 and 2.5 alleles per locus, respectively (Table 1). In the RLP site, nine private alleles were identified, representing almost 20% of all detected alleles, while in the DF stand, five private alleles were noted, accounting for approximately 12%. At the sex level, no private alleles were observed. In both stands, mean observed (H_O_) and expected (H_E_) heterozygosity were relatively low, with values below 0.50. The inbreeding coefficient (F_IS_) was low in the RLP population (F_IS_ = 0.055), whereas the DF population showed a substantially higher level of inbreeding, with a mean F_IS_ of 0.205. After correcting for the presence of null alleles, F_IS_ values in both stands were substantially lower, not exceeding 0.005. Null alleles (Null) were detected with an average frequency of nearly 7% in the RLP and approximately 8% in the DF population and were indicated as the likely factor of observed homozygosity excess (Table 1).

Significant sex-related differences in genetic diversity parameters were observed. In both stands, H_O_ (0.48 for males and 0.45 for females in RLP; 0.34 for males and 0.35 for females in DF) and H_E_ (0.51 for males and 0.48 for females in RLP; 0.39 for males and 0.42 for females in DF) differed significantly between males and females (*p* < 0.001; Table S2 in Supplementary File). In both populations, females exhibited a significantly (*p* < 0.001) higher level of inbreeding than males, with F_IS_ values of 0.042 vs. 0.033 at the RLP site, and 0.210 vs. 0.197 at the DF site. The observed heterozygote deficiency at the sexes level may result from the presence of null alleles rather than inbreeding. Null allele frequencies were similar between sexes in RLP (~6%), whereas in DF they were higher in females (8.3%) than in males (6.4%) (Table 1). Significant deficiencies in the M-ratio (*p* < 0.001) as a signal of bottleneck were detected in both stands and at the sex level (Table 1).

### 2.2. Clonal Diversity and Architecture

In the RLP population, 174 multilocus genotypes (MLGs) were identified among 501 sampled individuals, whereas in the DF stand, 153 genets were detected out of 568 trees. The probability of genotypes (P_gen_FIS) ranged from 4.08 × 10^−13^ to 1.10 × 10^−6^, with a mean value of 1.75 × 10^−8^ in RLP, and from 5.88 × 10^−10^ to 5.57 × 10^−5^, with a mean value of 5.20 × 10^−6^ in DF. The probability of encountering a genotype more than once by chance (P_sex_FIS) ranged from 0.00 to 7.47 × 10^−5^ (mean: 2.18 × 10^−6^) in RLP and from 6.44 × 10^−307^ to 5.77 × 10^−3^ (mean: 8.17 × 10^−5^) in DF. These low probabilities indicate that the repeated occurrence of identical genotypes within clustered MLGs is highly unlikely to result from independent sexual reproduction. Therefore, individuals sharing identical MLGs were considered clones (i.e., ramets of the same genet). In both studied sites, the number of MLLs (multilocus lineages) was equal to the number of MLGs.

Among the RLP genets, 96 were singletons (61 female, 35 male), whereas the remaining 78 genets were clones (36 female, 42 male). In the DF population, the detected genets included 77 singletons (39 female, 38 male) and 76 clonal genets (35 female, 41 male) (Table 2). No statistically significant difference in the number of female and male clones was found in both study sites (Z = −0.677, *p* = 0.498), indicating a balanced distribution of clonal genotypes between sexes. The genet size (NR) ranged from 2 to 45 ramets in the RLP population and from 2 to 43 ramets in the DF population.

At the population level, clonal diversity (R) was 0.346 in RLP and 0.269 in DF. Similar patterns of the Pareto distributions were observed in both sites, showing a skewed distribution of ramets within clonal lineages (Figure S3 in Supplementary File). The β values were 0.538 in RLP (r2 = 0.92, *p* < 0.05) and 0.603 in DF (r2 = 0.86, *p* < 0.05), indicating moderate evenness, with a few large genets and numerous smaller clones. Both populations exhibited high clonal heterogeneity, with the Simpson’s diversity index values exceeding 0.97 and the Pielou’s index above 0.86 (Table 2).

Significant sex-related differences in clonal richness were observed in the RLP population (*p* = 0.012), with females showing higher clonal diversity (R = 0.357) than males (R = 0.330). In contrast, no statistically significant difference was observed in DF (*p* = 0.431; Table S2 in Supplementary File). At both sites, the Pielou index differed significantly between sexes (*p* < 0.05; Table S2), with higher values recorded for males (RLP: 0.862 vs. 0.828; DF: 0.860 vs. 0.823). The Simpson’s diversity index did not differ between females and males in RLP, whereas in the DF site, significant sex-related differences (*p* < 0.001) were detected, with higher index values observed in males (D = 0.963) than in females (D = 0.949).

No biased sex ratio was detected at the genet (RLP = 1.26, p= 0.129; DF = 0.94, *p* = 0.686) and individual (RLP = 1.17, *p* = 0.081; DF = 0.97, *p* = 0.705) levels (Table S3 in Supplementary File). The intra-clonal distance to the nearest neighbour (dnear) ranged from 0.41 to 236 m in female clones (average 21.44 m) and 0.20 to 53 m in male clones (average 11.55 m) in RLP. In DF, females ranged from 0.47 to 336 m (mean 16.05 m) and males from 0.82 to 205 m (average 16.83 m) (Table 2). In both populations, spatial aggregation (A_c_) was consistently higher in females than in males, indicating that nearest-neighbour ramets of females more frequently belonged to the same MLL. In the RLP population, A_c_ values were 0.233 for females and 0.028 for males, while in the DF, the aggregation pattern was more pronounced with A_c_ = 0.639 for females and A_c_ = 0.458 for males (Table 2).

**Figure 1:**
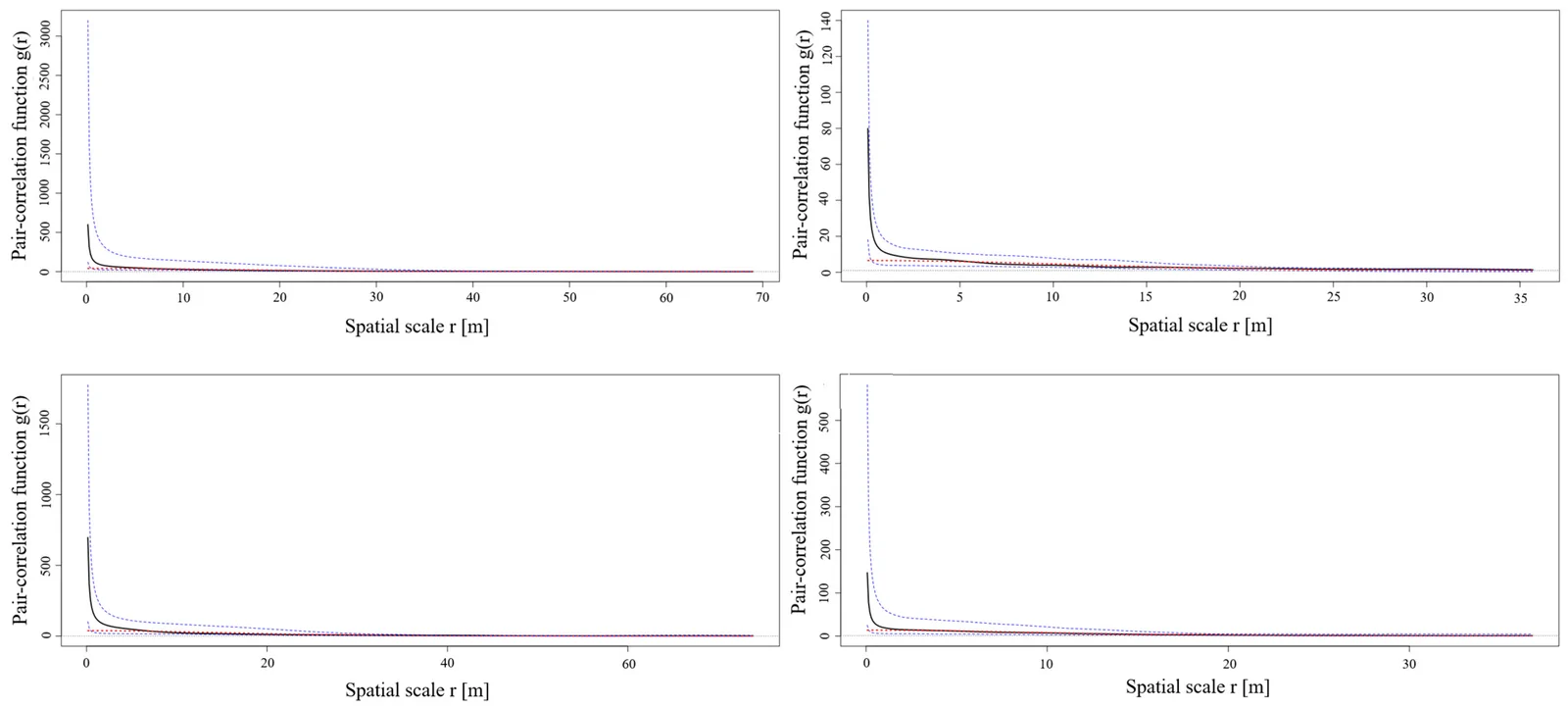
Pair correlation function g(r) plots for female (**upper panel**) and male (**lower panel**) ramets in the Rogalin Landscape Park (**right panel**) and Dębina Forest (**left panel**). Distance between ramets (r; in meters) and pair correlation function g(r), where g(r) = 1 indicates random distribution and g(r) > 1 indicates spatial aggregation; black solid line means g(r) observed, red dashed line means g(r) model and blue dashed line means 5% and 95% simulated.

The Thomas clustering process revealed clear patterns of spatial aggregation and highlighted sex- and site-specific differences in clonal architecture (Table 3). In the RLP populations, male clones contained more ramets per large cluster (μ_1_ = 38.5) than female clones (μ_1_ = 34), and also male clusters had a larger span (2σ_1_ = 54.049 m vs. 30.485 m in females). In contrast, within small clusters, females exhibited both a higher number of ramets per cluster (μ_2_ = 19.28) and a larger cluster diameter (2σ_2_ = 28.209 m) compared with males (μ_2_ = 16.5, 2σ_2_ = 28.018). Male clones in the RLP exhibited a somewhat more aggregated architecture, reflected by a slightly higher clonal dominance index (D_C_ = 0.796) compared with females (D_C_ = 0.721). In the DF, male clusters were also larger in terms of ramet number (μ_1_ = 48 vs. 35 in females), but slightly more compact, with a smaller cluster radius (2σ_1_ = 31.028 m vs. 2σ_1_ = 33.591 m). In small clusters, females formed clusters with more ramets and exhibited a higher cluster diameter (μ_2_ = 16.4, 2σ_2_ = 25.114) than males (μ_2_ = 14.4, 2σ_2_ = 17.583). As in RLP, males also showed a higher clonal dominance index (D_C_ = 0.719 vs. 0.659) in DF. The observed g(r) confirmed these patterns, showing strong aggregation at short distances in all stands and both sexes (Figure 1), expressed by a peak at short spatial scales, exceeding the expectation of spatial randomness (g(r) = 1). The absence of systematic deviations at larger distances suggests that aggregation was primarily driven by clonal clustering.

### 2.3. Spatial Genetic Structure

**Figure 2:**
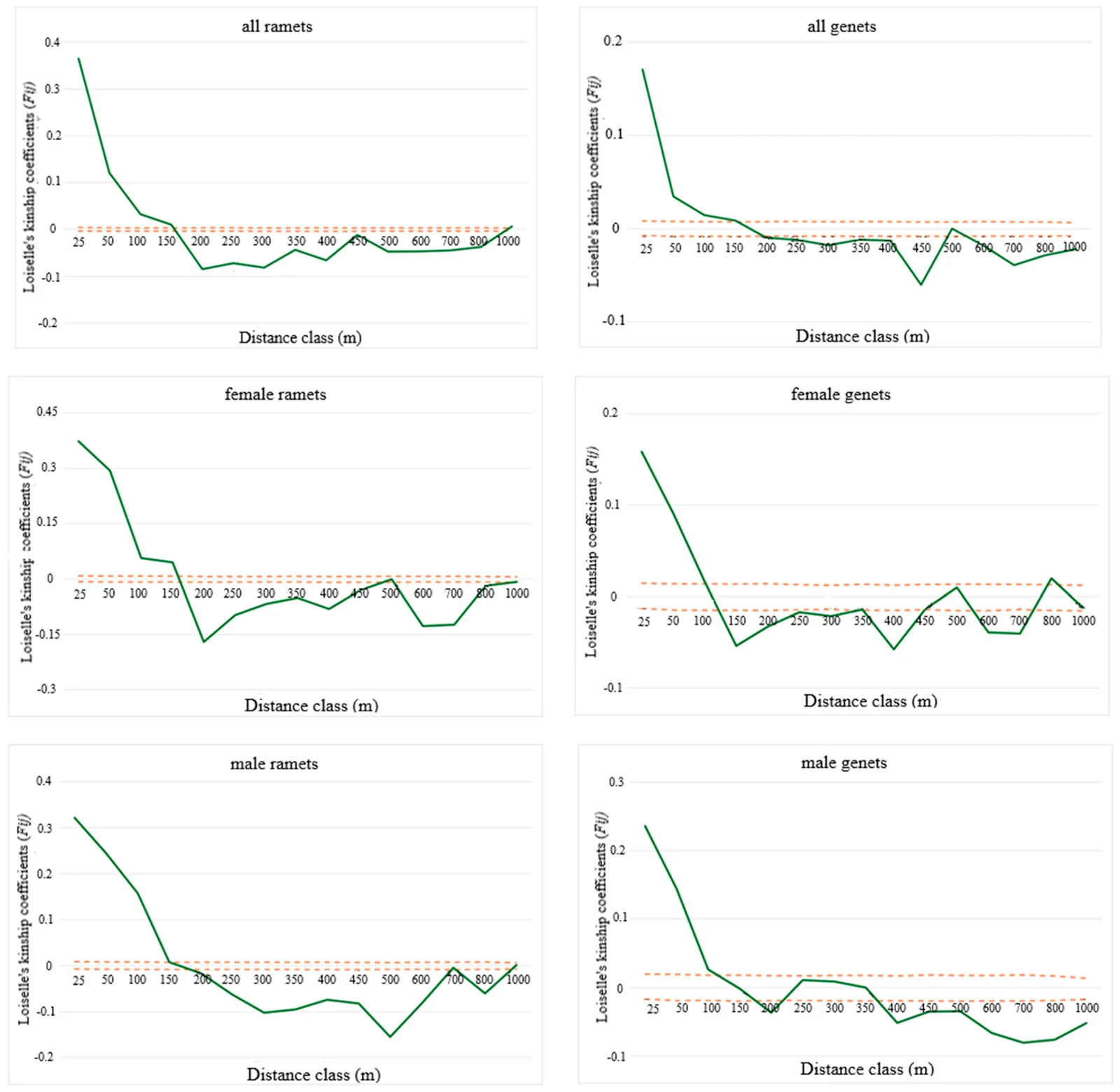
Kinship coefficients (F_ij_) plotted against the maximum distance between individuals in each distance class defined for the Rogalin Landscape Park. Dashed lines show the limits of the 95% confidence interval around the null hypothesis based on permutation tests.

**Figure 3:**
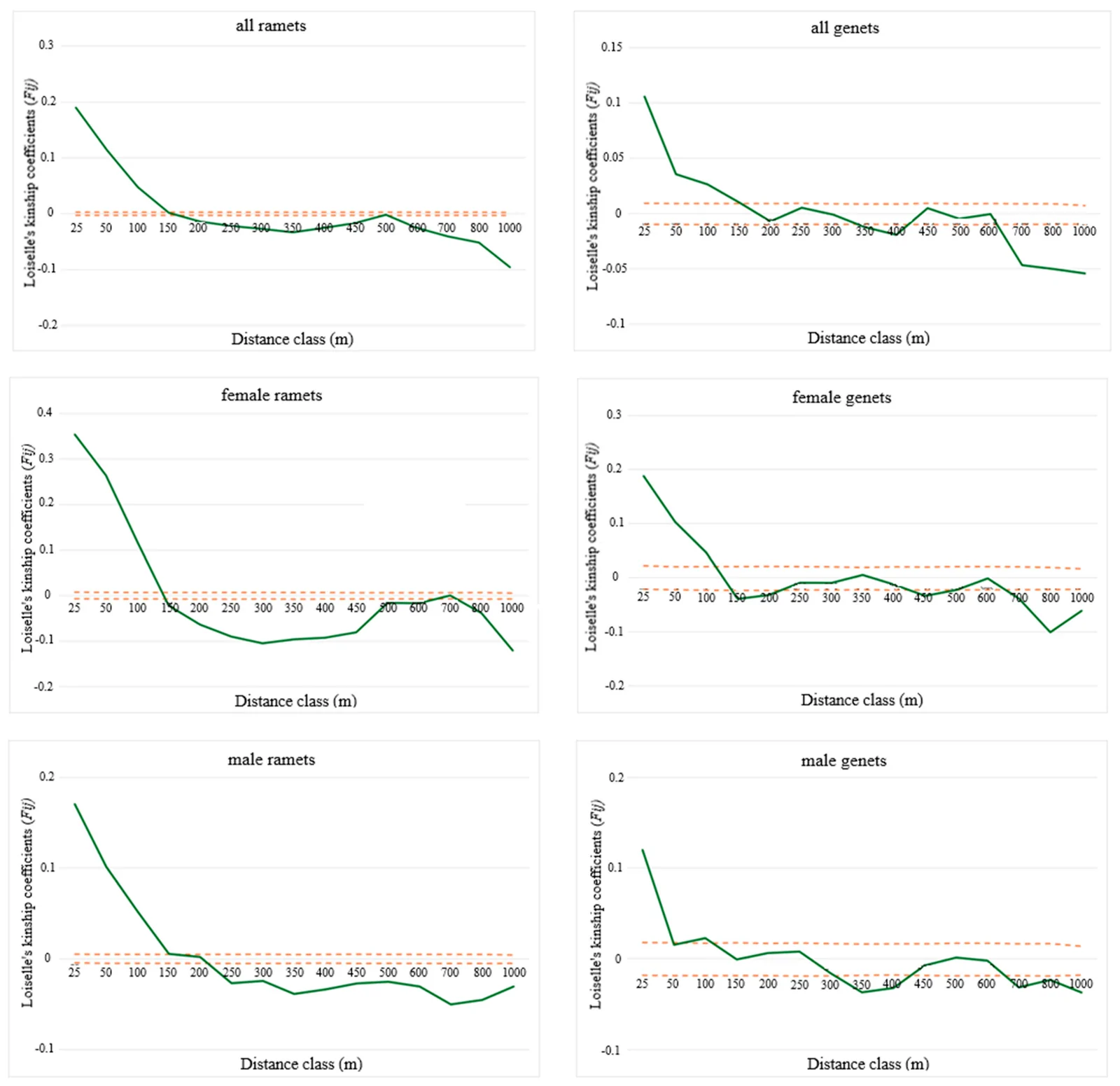
Kinship coefficients (F_ij_) plotted against the maximum distance between individuals in each distance class defined for the Dębina Forest. Dashed lines show the limits of the 95% confidence interval around the null hypothesis based on permutation tests.

Similar patterns of spatial genetic structure were observed across stand and sex levels between sexes (Figure 2 and Figure 3). The regression slopes (b_F_) were consistently negative and significant, ranging from −0.027 to −0.065 at the genet level and from −0.055 to −0.115 at the ramet level (Table 4). At the ramet level, the significant positive average kinship coefficients (F_ij_) were noted in the first distance classes up to 150 m in females in both stands. In males, F_ij_ remained positive up to 150 m in RLP and up to 200 m in DF. The mean F_ij_ did not consistently decrease with increasing spatial distance, and significant positive values were also observed at 1000 m for male ramets in RLP and at 700 m for female ramets in DF (Figure 2 and Figure 3). At the genet level, significant positive F_ij_ was also observed in the shortest distance classes up to 150 m in both sites, and this pattern was consistent for both females and males (Figure 2 and Figure 3). Beyond 150 m, F_ij_ values were generally negative, except for a significant positive F_ij_ recorded at 800 m for female genets in RLP (Figure 3).

Overall, the Sp values tended to be higher for ramets than for genets across both stands and sex levels. On average, Sp values at the ramet level were approximately twofold higher than those at the genet level (Table 4). Among ramets, Sp values were higher for females than for males, being nearly fourfold higher in RLP and more than twofold higher in DF. However, the overall SGS intensity at the ramet level was lower in DF than in RLP. No substantial difference in Sp values was observed between male genets and ramets in either stand. Among genets, over twofold higher values of Sp were noted in males than in females in the RLP, whereas the opposite pattern was observed in the DF (Table 4).

### 2.4. Population Genetic Substructure

**Figure 4:**
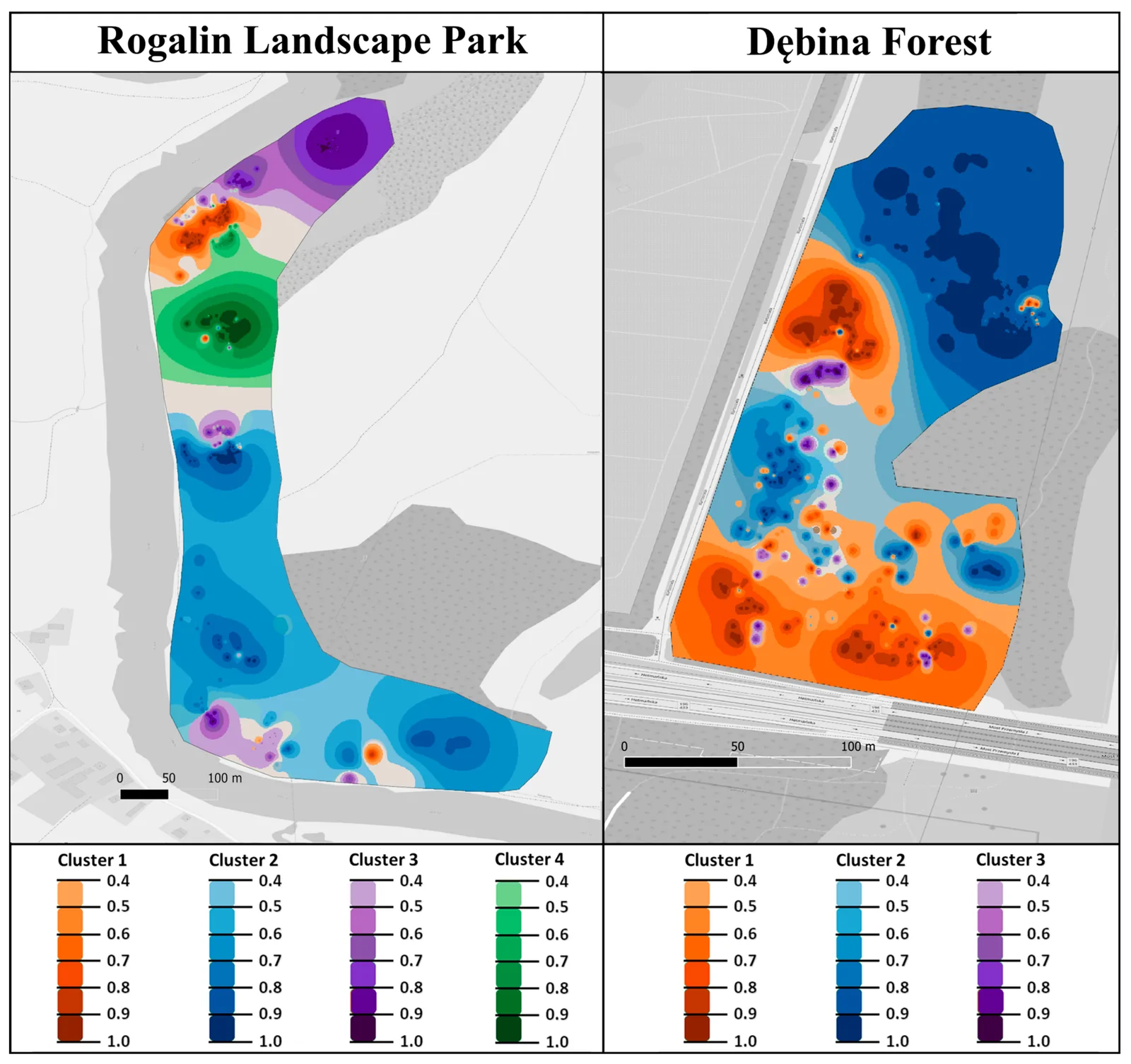
Spatial genetic structure of white poplar stands based on STRUCTURE results at the genet level. The substructuring among individuals was shown at K = 4 for the Rogalin Landscape Park and at K = 3 for the Dębina Forest. The colour scale represents the probability of assignment of each individual to the inferred K cluster.

According to Structure, an optimal number of clusters for both stands for data not divided by sex was K = 2 (see Supplementary File, Figure S4); all genets were divided into two distinct genetic clusters with relatively high admixture. Furthermore, the second-best number of clusters was K = 4 in the RLP, and K = 3 in the DF stands, indicating the hidden substructure among individuals reflecting sex-dependent patterns (Figure 4). The probability of assignation of individuals to the inferred genetic clusters was relatively comparable in both stands, indicating also a high level of genetic admixture (Supplementary File, Table S4). A closer inspection of this substructure revealed that clusters differed in sex composition and the proportions of clones and singletons (Supplementary File, Table S5).

**Figure 5:**
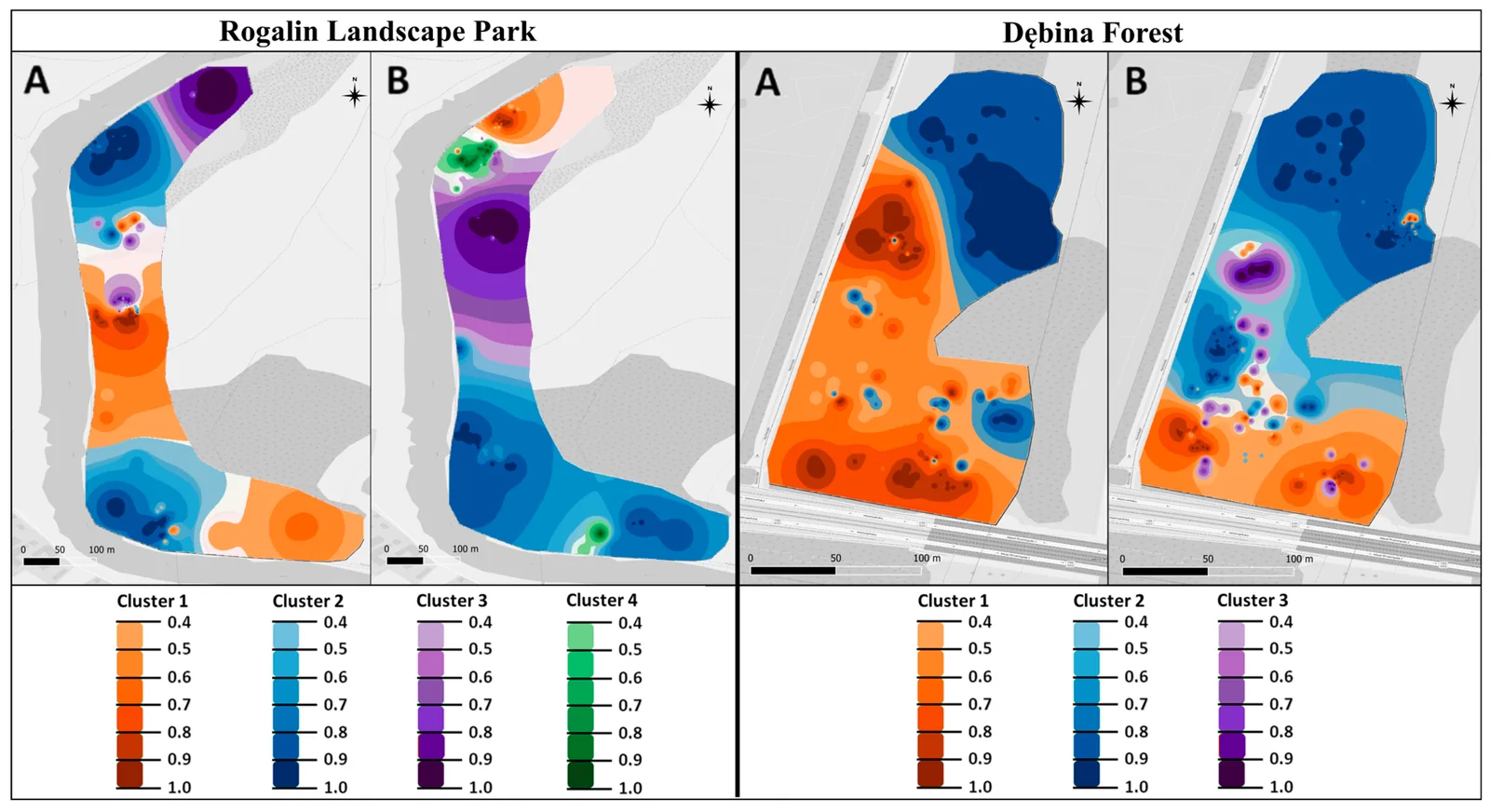
Spatial genetic structure of white poplar stands at the sex level ((**A**)—female, (**B**)—male) based on STRUCTURE results. In the Rogalin Landscape Park, substructure was detected at K = 3 for females and K = 4 for males, whereas in the Dębina Forest it was observed at K = 2 for females and K = 3 for males. The colour scale represents the probability of assignment of each individual to the inferred K cluster.

The additional genetic clustering, conducted separately by sex in both stands, confirms substructuring among female and male individuals. In the RLP, female individuals were grouped into three clusters (K = 3), whereas males were grouped into four clusters (K = 4) with a moderate level of genetic admixture. In the DF, female individuals were assigned to two clusters (K = 2) with relatively similar mean membership coefficients, whereas males were assigned to three clusters (K = 3), also with a moderate level of genetic admixture. Details on the proportion of individuals assigned to each inferred genetic cluster are provided in Table S6 (Supplementary File). Although a high level of admixture within each sex was observed, the spatial distribution of genetic groups mirrored the spatial aggregation of individuals across the landscape (Figure 5).

## 3. Discussion

Spatial genetic structure has profound ecological and genetic consequences in plants, and clonality is increasingly recognised as a substantial driver of SGS, also in tree species [51,52,53]. At the same time, it has been recognised that dioecy may also leave a distinct imprint on SGS [9,12]. However, our understanding of how clonality and dioecy jointly affect SGS in long-lived trees remains incomplete, and we address this question in this study. Our results strongly support the hypothesis that spatial dependence in sex distribution, reinforced by clonality, acts synergistically to enhance SGS patterns in white poplar. We can conclude that sexual dimorphism in clonal trees may affect clonal architecture and the local distribution of male and female individuals, which in turn influences effective gene dispersal. We discuss how clonal diversity and clonal architecture contribute to sex-specific SGS patterns in white poplar, with habitat context considered as an additional factor influencing their expression. Particular attention is given to the role of disturbance patterns and habitat conditions in shaping the balance between sexual and asexual reproduction, as well as the implications of these processes for the management of genetic resources in riparian tree species.

### 3.1. Clonal Diversity in White Poplar

Our study indicates that clonal reproduction is the predominant growth strategy at both white poplar sites. The average value of genotypic diversity for 195 different clonal plant taxa (terrestrial and aquatic) is 0.42 [2], while the estimation for tree species provided by Silvertown [54] revealed a value close to 0.40. Although lower than average for trees, clonal diversity estimated for our populations of white poplar considerably exceeds those reported for other stands (R = 0.13; [5]; R = 0.138; [44]; R = 0.163; [43]). The comparatively higher clonal diversity observed in our case may reflect differences in demographic, genetic, and environmental factors that influence the balance between sexual and asexual reproduction in partially clonal species [2,9,53,55,56,57,58,59]. Among environmental factors, habitat conditions are of critical importance for poplars. These species produce abundant, tiny, non-dormant seeds that disperse over long distances by wind and water; however, their recruitment depends on specific conditions because the seeds are extremely short-lived [60,61]. The regeneration mode in white poplar is linked to the fluvial geomorphic events (erosional and depositional processes) occurring in the floodplain forest and creating new nursery sites suitable for seed germination and seedling establishment [62,63,64]. Periodic cycles of floods remove competitors and create a bare and moist substrate available for immediate colonization. Therefore, the critical overlap of flood events and seed release time and duration creates a “window of opportunity” permitting sexual regeneration [62]. In populations occurring in suboptimal habitats, factors such as resource limitations, especially insufficient moisture, and a disturbed fluvial regime may limit successful sexual recruitment, leading to increased asexual input. The low clonal diversity in the population studied by Dering et al. [9] was driven by the lack of habitats suitable for sexual recruitment due to changed flood dynamics resulting from dam construction and river flow regulation. In both populations studied here, sporadic flooding is likely to occur and supports sexual recruitment, as indicated by higher clonal diversity indices. Accordingly, Silvertown [54] stated that lower frequency or absence of disturbances, which are periodic floods in the case of floodplain forests, results in the predominance of asexual over sexual reproduction. Therefore, among-population variation in clonal diversity should primarily be interpreted in the context of flood dynamics and frequency, as these processes are critical for generating recruitment sites and new genotypes emerging, which all models of clonal diversity predict [64].

Furthermore, low clonal diversity may reflect the negative effect of senescence on sexual reproduction, resulting in a lack or low contribution of sexual progeny [65,66,67]. As demonstrated in *Populus tremuloides* [65], the accumulation of somatic mutations in ageing male clones impacts fitness by impairing pollen production, which may reduce fertility. The senescence of clonal individuals may partially explain the dominance of asexual reproduction, as in the case of the Sardinian populations of white poplar [43]. In the studied populations, the dendrochronological age of the oldest male clones was 145 years in the Rogalin Landscape Park and 78 years in Dębina Forest. However, the true age of the respective genotypes remains unknown since the exact time of seedling (clone) establishment is rarely known, especially in trees [68]. Hence, the genetic turnover in clonal trees is impossible to measure due to their longevity. The dendrochronologically-estimated age of currently present ramets in the population may not reflect the biological age of the clones [65,69].

The clonal diversity of a population may vary with age due to among-clone competition and stochastic and demographic mortality [68,70]. Unless colonisation is accompanied by a severe founder bottleneck, the initial seedling cohort should comprise a genetically diverse pool of individuals, yet with varying capacities for clonal reproduction [71,72]. In partially clonal species such as white poplar, differing ability of genotypes to clonal spread typically manifests as co-occurrence in a population of singletons and multi-ramet individuals [9,73,74,75]. As individuals grow and the stand expands, resource pre-emption intensifies among-clone competition, leading to the loss of genets *via* demographic and competitive thinning. If sexual recruitment is absent or infrequent, clonal diversity is expected to decline progressively through time, leaving the most proliferous and best-adapted clones [2,9].

Regarding genet size distribution, both studied stands showed a skewed Pareto distribution, which is a typical size distribution pattern in clonal plants [73]. However, in the studied populations, the shape parameter of the Pareto distribution (β) was half of that noted in other poplar populations (1.07 in *Populus nigra*; [8] and 1.35 in *Populus alba*; [5]). Consequently, in our stands, large (>20 ramets) and medium-sized clones (>5 ramets) constitute a considerable fraction of the distribution of clone size, pointing to the importance of clonal propagation of once established genotypes. Besides the genotypic propensity to produce clonal progeny [44,72], suckering in species like poplars can be a result of a disturbance-related condition of the root system following injury or stress, which is in accordance with the persistence strategy (persistence niche), typical for species from disturbance-prone ecosystems [70,76]. The dynamics of disturbance may affect the level of clonality in a stand generally, but it may also lead to highly localised clonal expansion through sprouting from flood-trained roots [77]. Both populations studied here are under different spatio-temporal levels of natural and anthropogenic disturbances, which may explain the slight differences in clonal diversity between them. In the congeneric species *P. nigra*, the level of clonality increases as anthropogenic transformation of river systems intensifies [75]. In the Rogalin Landscape Park, the white poplar population is located in a semi-natural, agricultural landscape in the direct vicinity of the arable fields. Mowing, ploughing, and sporadic pasture, which are all noted in this stand, may be responsible for higher suckering. While a catastrophic disturbance like floods may facilitate turnover of genets and recruitment from seeds, mild disturbances may induce suckering [70,78].

### 3.2. Clonality and Spatial Genetic Structure

The spatial aggregation of clonal progeny increases SGS in the same way as clustering of full sibs in non-clonal species with restricted seed dispersal mode [9,79,80,81]. The immediate effect of clonality on SGS is detected at the ramet level and short-distance classes [5,8,22,81,82]. Regarding the magnitude of SGS in clonal species, the spatial arrangement of clonal progeny (the clonal architecture) is an important factor [2,9].

The elevated Pielou index (J > 0.86) for our populations indicates that ramets of the same genet tend to occur in proximity, forming distinct clonal patches and indicating limited mixing of multilocus lineages (MLLs). Elevated values of the aggregation index (A_c_), especially in the Dębina Forest, indicate that nearest-neighbour ramets more often belong to the same genet than expected by chance, while higher clonal dominance index (D_c_) values point to a more compact clonal architecture. In both populations, the maximum distance between ramets of the same genet exceeded 200 m, while the average nearest-neighbour distance was less than 25 m, showing a generally compact clonal architecture with occasionally very distant vegetative spread. The long-distance genotypic matches detected in our stands are likely due to the hydrochorous movement of vegetative fragments [70,83]. The spatial characteristic of the clonal spread in the studied stands indicates a *phalanx* growth described for white poplar [5,9] and for other poplar species [8,70,71,84]. The *phalanx* growth strategy may limit the intermixing of genets, resulting in greater SGS in clonal plants at the ramet level [4,14,9,85].

Our findings demonstrate that clonality markedly strengthens fine-scale spatial genetic structure at the ramet level. This observation aligns with previous research on single populations of the species [5,23] and other clonal plant species [73,81,82,85,86]. Specifically, the SGS intensity, assessed using *Sp* statistics, was almost twofold stronger at the ramet level than at the genet level in both stands (RLP: 0.116 vs. 0.045; DF: 0.085 vs. 0.047). Furthermore, ramet-level SGS was considerably more pronounced in the semi-natural RLP habitat (*Sp* = 0.116) compared to the urban DF habitat (*Sp* = 0.085). While both populations exhibit a *phalanx* growth strategy, their clonal architecture differs significantly. In the Dębina Forest, the spatial pattern of clonal spread indicates much stronger clumping, relative to the Rogalin Landscape Park. Clonal architecture is a plastic trait that enables clonal plants to optimise individual ramet and whole genet performance in habitats with patchily distributed resources [87]. This architecture is primarily shaped by variability in resource access, the number and size of resource patches, and competitive interactions [88]. Accordingly, site heterogeneity in the studied stands likely drives the strategy of new ramet placement, consistent with the foraging behaviour and division of labour predicted for clonal species [87,89,90]. Comparing the average number of individuals per cluster, the range of ramet grouping in the urban area (DF) was greater in the large cluster, while lower in the small cluster than in the semi-natural RLP stand. This suggests that either the increased anthropogenic pressure (e.g., mowing) affects clonal recruitment [91] or the network of walking pathways limits ramet survivorship. Additionally, neither stand represents an active alluvial zone, and competition from gradually infiltrating later-successional species, such as *Alnus glutinosa*, *Fraxinus excelsior*, *Ulmus laevis*, and *Quercus petraea*, may further impact clonal spread by interspecies competition. This environmentally driven clonal architecture would, in turn, shape the magnitude of ramet-level SGS. However, to directly link habitat quality and clonal architecture, a more comprehensive stand analysis, including vegetation cover heterogeneity, soil properties (e.g., moisture, N and K concentrations), light heterogeneity, and competitor basal area, would be required.

Consistent with previous findings [5], the spatial aggregation of related genotypes in the studied white poplar stands produced significant SGS at the genet level. The strength of SGS in clonal plants should first be interpreted considering clonal diversity and its temporal dynamics. In partially clonal species, clonal diversity usually decreases over time as asexual reproduction becomes more prevalent, and a few persistent genets may dominate the local population [70]. In both white poplar stands examined here, clonal diversity was low, implying restricted mating opportunities; under such conditions, cumulative local ancestry and repeated local recruitment are expected to reinforce SGS. Gene flow can mitigate SGS; however, in riparian poplar, it is frequently restricted by fragmentation of floodplain forests and hydrological alterations that reduce recruitment and, consequently, effective gene flow [9,75,92]. Although the level of gene flow is unknown for the studied population, our indirect results of gene dispersal clearly suggest restriction of genetic connectivity. A second, complementary mechanism enhancing SGS in clonal species derives from reproductive success. In clonal, dioecious populations, clone-size distributions are often leptokurtic, which may generate high variance in male and female fecundity and thus reduce the effective number of parents contributing to local pollen and seed pools [2]. When a few individuals disproportionately contribute to the available gamete pool for mating, correlated paternity/maternity increases. Dering et al. [9] reported extensive sibship structure in white poplar, with half-sib relationships detected for 33% of genets, which is consistent with the elevated SGS observed. Pospíšková and Šálková [93] also indicated domination of within-population pollinations in the black poplar stands studied, with 77% of offspring being fathered by males from the local population. However, with the present study design, we cannot verify the role of local mating and restricted gene flow in generating significant SGS in the studied populations.

### 3.3. Dioecy and Spatial Genetic Structure

Differences in clonal architecture between female and male individuals may lead to sex-specific spatial patterns and influence population structure [33]. The dissimilarities may derive from sex-specific strategies of resource allocation and fitness of genets [2,18]. Our findings revealed divergent patterns in clonal architecture in male and female genets, although they differed depending on the population studied and parameter considered. The clonal richness (R) was significantly higher in females than males in the Rogalin Landscape Park (p = 0.012), yet no such difference was found in Dębina Forest, suggesting that site-specific conditions drive recruitment and/or mortality of male genets.

In contrast, males consistently exhibited greater Pielou index values, signifying that ramets were generally more evenly distributed among genets. The aggregation index (A_c_) further supported differences, as females had greater values than males (RLP: 0.233 vs. 0.028; DF: 0.639 vs. 0.458), demonstrating that female ramets were more likely to occur proximate to ramets of the same genet. In both sites, female genets extended over greater distances (RLP: 236.75 m; DF: 336.51 m) as compared to male genets (RLP: 53.28 m; DF: 205.64 m). Further differences in spatial dependence in the occurrence of sexes emerged from the Thomas clustering model and clonal dominance index (D_c_), which indicated opposing patterns of local dominance and clone intermingling between sexes and populations. In both sites, males tended to form clusters composed of more ramets and exhibited slightly higher clonal dominance than females (RLP Dc = 0.796 vs. 0.721, DF Dc = 0.719 vs. 0.659), indicating a more compact structure and limited overlap of genets. Although female clones showed greater spread based on distance to the nearest ramet of the same clone (d_near_), especially in the RLP site, our results suggest that males have more concentrated clonal architecture, reflected in a greater proportion of ramets belonging to the same genet within clone-defined areas. Similar findings were reported by Liao et al. [94], who showed that although male and female plants of *Acer barbinerve* occurred under comparable environmental conditions in the same population, they exhibited sex-specific responses in growth and reproduction, reflecting different adaptive strategies.

To assess whether observed sex-specific clonal arrangements translate into differences in genetic structure, we examined spatial genetic structure (SGS), with a particular focus on the possibility of sex-dependent SGS. In both populations, sex-specific differences in spatial genetic structure were observed, with females exhibiting stronger SGS than males. These findings support the idea that dioecy affects the spatial distribution of genotypes [12]. This was evident in both populations, where female ramets had higher *Sp* values (RLP: 0.129; DF: 0.177) than male ramets (RLP: 0.115; DF: 0.066). The same trend was reflected in the kinship coefficient at the first distance class (F_ij(25m)_), because in both populations at the ramet level, females had a higher F_ij_ than males. Interestingly, this pattern is opposite to previous findings in *P. alba*, where stronger SGS was observed in male individuals, although not at the ramet level [9]. The female-biased SGS may be due to differences in life history strategies between the sexes. Typically, female individuals allocate energy into seed production, probably at the expense of vegetative propagation and spread capacity [18,95,96]. Environmental conditions also play an important role, as moisture- and nutrient-rich sites may mitigate stress-induced mortality among females, which are generally more sensitive to resource limitations [24,97].

The observed differences in SGS between sexes may also relate to broader spatial patterns within populations. One such phenomenon is spatial sex segregation (SSS), which may arise in dioecious and clonal species due to differences in clonal spread, recruitment, or microhabitat preference [9,98]. In our study, clustering analysis revealed the presence of a different number of genetic groups for each sex in the two populations. In both populations, males and females exhibited high admixture levels, while sex-specific differences were expressed as subtle shifts in cluster membership patterns. The slight differences in population substructure suggest that migration and recruitment mechanisms may follow different patterns depending on sex [32], which, for dioecious plants, has been confirmed in other studies [9,17,30].

### 3.4. Genetic Diversity: Implications for the Management of Genetic Resources

We observed overall low genetic diversity for both studied stands of white poplar (on average H_E_ = 0.45, A_R_ = 2.589; Table 1). These values are consistent with levels reported for other European populations at fine scale (H_E_ = 0.376 in [23]; H_E_ = 0.387, A_R_ = 3.25 in [99]), as well as detected at regional scale for Iberian marginal populations (H_E_ = 0.456, A = 2.40; [44]) and Turkish stands (H_E_ = 0.460, A = 3.41; [47]). However, the detected level of diversity is lower than that reported for other Polish populations (H_E_ = 0.622, A = 6.310; [5]) and isolated stands in Sardinia (H_E_ = 0.60, A = 5.0; [43]). This pattern is not unexpected, given the patchy distribution of the species due to habitat fragmentation, impeding genetic connectivity, and the reduced potential for sexual regeneration or the impact of clonal growth on mating.

Over the last two centuries in Poland, a 95% decline in riparian forests has been reported due to river regulation and hydrotechnical modifications [100]. The anthropogenic changes disrupt the natural hydrological regimes required for the “windows of opportunity” recruitment strategy, which drives episodes of sexual recruitment in floodplain forest species [5]. We detected signs of recent bottlenecks in both studied stands, suggesting that adverse demogenetic processes contributing to genetic erosion are already underway. Similar genetic depletion was also observed in black poplar along the extensively transformed section of the Warta River in Poland [75,100]. This shared pattern in both species reflects broader regional environmental pressures operating within the Warta River valley, which in turn often result in decreased effective population size and intensified SGS. Although occasional sexual recruitment was observed in the studied stands, the detected low genetic diversity likely results from limited natural regeneration under ongoing agricultural practices, which hinder seedling establishment and survival, favouring clonal persistence.

Despite aggregated growth reinforcing fine-scale SGS and increasing the likelihood of mating among relatives [9,67], we observe low inbreeding in the studied populations, consistent with Dering et al. [5]. However, FIS values reported here refer to adult trees rather than offspring cohorts, in which the negative effects of mating limitation imposed by clonality may be more pronounced, as cumulative inbreeding can be detected only in offspring. Crucially, monitoring of progeny generations should be prioritised to obtain a deeper insight into genetic trends.

The management and conservation of genetic resources in clonal trees, such as white poplar, present significant challenges due to the spatial aggregation of ramets and the cryptic nature of clonal structures. Such challenges are amplified in dynamic riparian systems, where flood patterns influence clonal turnover and shift the sexual-asexual balance, complicating effective conservation planning. To properly establish ex-situ conservation collections and select propagation material, sampling schemes should account not only for spatial proximity among individuals but also for sex-specific clonal aggregation. Given that individuals within 100–150 m are often closely related, clonal spread can exceed 200 m, and SGS may remain significant up to 300 m [5,23,9], we recommend collecting plant material from individuals spaced at least 200–300 m apart to minimise the risk of oversampling the same genets at the local scale. Even such a distance-based guideline should be applied cautiously, because of extensive clonal spread of the species and formation of massive monoclonal stands [43,44]. These differences in sex-specific clonal architecture have direct implications for conservation and restoration, pointing to the need for sex-specific stratified sampling approaches and wider spacing between sampled female individuals than between males. This interpretation is further supported by genetic clustering analyses, which revealed a clear sex-dependent spatial structure, implying that a single, systematic sampling approach within spatially separated female patches may be insufficient. Ultimately, in populations exhibiting high SGS, sampling should be stratified across identified genetic clusters rather than relying on spatial distribution alone, thereby ensuring a more representative capture of the underlying genetic diversity.

In cases where genetic characterisation is not feasible, this distance-based approach may be cautiously complemented by collecting material from several spatially close populations within the same region or river basin, preferably located along weakly regulated or near-natural river sections. Such an approach may increase the probability of capturing a broader range of genotypes, particularly where individual populations are small, spatially restricted or strongly clonal [101,102]. This is consistent with EUFORGEN recommendations for white poplar, which suggest including more than one conservation site per river system and recommend preliminary genetic assessment to conserve genetic diversity while limiting clonal duplicates [37]. It is also broadly consistent with multiple-source strategies in conservation translocations, where material from several populations may increase genetic diversity and reduce the risk of inbreeding, provided that the risk of outbreeding depression is considered low (IUCN/SSC 2013). The mixing of material from several populations should therefore be carefully documented to reduce the risk of uncontrolled genetic homogenisation and the loss of local genetic structure [103]; IUCN/SSC 2013].

## 4. Materials and Methods

### 4.1. Study Site and Sampling

**Figure 6:**
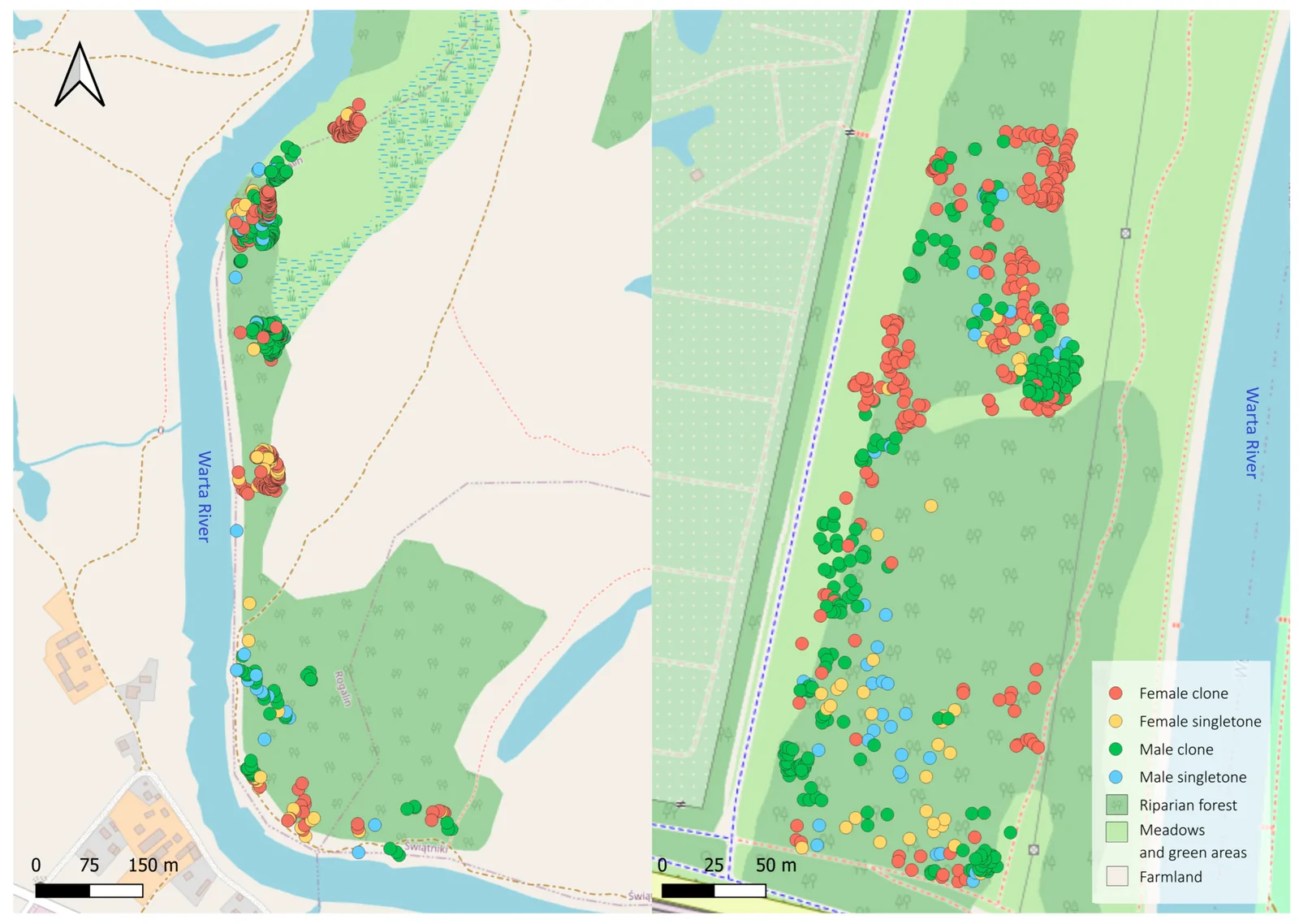
Study sites of white poplar and sampling scheme in the Rogalin Landscape Park (**left**) and Dębina Forest (**right**). Map generated in QGIS 3.44 using OpenStreetMap base layer (Qgis.org 2024).

To investigate the processes driving spatial genetic structure in white poplar, we selected two stands along the Warta River in Poland representing different landscapes with contrasting disturbance regimes: Rogalin Landscape Park (RLP) (Rogalin; 52°13′01′′ N, 16°55′28′′ E), a semi-natural site, and Dębina Forest (DF) (Poznań; 52°23′01′′ N, 16°56′02′′ E), an urban stand (Figure 6).

Sampling was conducted in the spring and summer of 2022. At the RLP site, which covers an area of 10.5 ha, 501 individuals (ramets) were sampled, while 567 individuals were recorded at the DF stands, which span 2.5 ha (Figure 6). In the RLP site, the distribution of individuals has a clumped (clustered) arrangement, forming dense groups located along the river. In contrast, in the DF site, trees are separated from the river by green areas and walking paths, resulting in a more random distribution pattern. For RLP, the tree density was 21.53 individuals per hectare, while for DF it was 154.5 individuals per hectare.

To estimate the ages of the studied stands and clones, core samples from approximately 65 trees per study site (two samples per tree; in total 260 samples) at 120 cm high with the Pressler increment borer were collected. Within each study site, all trees ≥ 150 cm in height were sampled and georeferenced using a Spectra Precision GNSS SP60 geodetic receiver (Trimble Agriculture, Westminster, CO, USA). The trees’ height was measured using the Vertex IV and Transponder T3 (Haglöf, Långsele, Sweden). Sex was identified based on direct observation during the flowering period in 2023 and further confirmed in the next season. Non-flowering or immature individuals were sexed based on the genotypically determined sex of the clone to which they were classified. Individuals that were non-flowering singletons or belonged to a clone with undetermined sex were excluded from the analysis.

### 4.2. DNA Extraction and Genotype Acquisition

Total genomic DNA was extracted from leaf tissue collected from 1068 individuals according to the protocol described by Dumolin et al. [104]. Genotyping was performed with a set of 15 nuclear microsatellite markers (nSSRs) developed for *Populus nigra* by Tuskan et al. [105] and van der Schoot et al. [106]. The markers were assembled into the following multiplexes: Multiplex I—WPMS5, WPMS6, WPMS14, WPMS15; Multiplex II—ORPM30, ORPM312; Multiplex III—ORPM149, ORPM186, ORPM190, ORPM214; Multiplex IV—WPMS10, WPMS12, PMGC2852; Multiplex V: ORPM21, ORPM28. Details on nSSRs used for genotyping and PCR conditions are provided in Table S1 (Supplementary File).

Fluorescently labelled amplification products were separated using an ABI PRISM 3130XL^®^ automated capillary sequencer (Thermo Fisher Scientific, Waltham, USA) with an internal size standard, GeneScan™ 600 LIZ^®^. The fragments’ sizes were scored using GeneMapper 4.0 (Thermo Fisher Scientific, Waltham, MA, USA), manually verified, and binned using Raw2Gen software [107].

### 4.3. Clonal Assignment

The identification of unique multilocus genotypes (MLGs) and multilocus lineages (MLLs) was done using the RClone R package, v.1.0.3 [108] (R Core Team 2024). First, we discriminated the MLGs with the MLG_tab function; then, we performed a test for the reliability of the used loci and subsampling of individuals, using the sample_loci function. To avoid biases arising from varying sampling sizes across studied populations, the expected MLG (eMLG) based on the rarefaction method was estimated with the mlg.table function implemented in the poppr R package, v.2.9.6 [109].

To minimise misclassification of MLLs, we accounted for merging error, in which similar genotypes are incorrectly identified as clones, and splitting error, which occurs when dissimilar fingerprints from clones are incorrectly identified as genetically separate individuals [73,110]. Accordingly, the probability of occurrence of each observed genotype under sexual reproduction with random mating (P_gen_ F_IS_) using the function pgen_F_IS_ and the probability that the similar genotypes are a result of independent sexual reproduction events (P_sex_ F_IS_), using psex_F_IS_, were estimated. As recommended by Arnaud-Haond et al. [73], P_gen_ estimation was adjusted for departures from the Hardy–Weinberg equilibrium (HWE), using F_IS_ values achieved using allele frequencies assessed by the Round-Robin method, a sub-sampling approach which avoids overestimation of rare alleles [8,111]. If P_sex_F_IS_ < 0.01, we concluded that individuals classified into a given MLL were really clones of a given genet. To verify this classification and reduce the risk of splitting errors, we also used the MLL_generator function. Finally, we checked the match between individuals sharing the same genet in terms of gender and spatial distribution.

### 4.4. Genetic Diversity

The deviation from the HWE for each population and locus was tested using the exact test implemented in GENEPOP v.4.4 [112]. Further, we tested linkage disequilibrium for loci using the log-likelihood ratio statistic test for each pair of loci with Bonferroni correction (Supplementary file) in GENEPOP. We assessed the presence of null alleles (Null) and genotyping failure rate in the final dataset using INEst v.2.3 [113].

Genetic diversity parameters at the genet level were assessed separately for each sex and population using the number of alleles per locus (A), observed (H_O_) and expected heterozygosity (H_E_), private alleles (Ap) and inbreeding coefficient (F_IS_) estimated in INEst. Allelic richness was calculated using the rarefaction method implemented in the function allelic.richness from the hierfstat R package, v. 0.5-11 [114] based on the smallest sample size across populations. The inbreeding coefficient (F_IS_) was estimated including null alleles correction (F_IS_Null), using the Individual Inbreeding Coefficient model implemented in INEst [113]. The significance of the inbreeding was evaluated by comparing the full model with the random mating model (i.e., when *F_IS_* is 0) based on the Deviance Information Criterion (DIC). The calculations were run using 5 × 10^5^ MCMC iterations with sampling every 200 iterations and a burn-in of 5 × 10^4^. To test statistical differences in mean H_O_, H_E_ and F_IS_ values between sex and study sites, the Z-test was applied, as recommended for large sample sizes [115]. Statistical significance was evaluated at α = 0.05 (Supplementary file).

Evidence of a recent bottleneck in studied populations was assessed using the M-ratio test [116] and the heterozygosity excess method [117] implemented in INEst. The observed MR values were tested against the demographic equilibrium value MR_eq_ using the Wilcoxon signed-rank test. The M-ratio (MR) was estimated based on the coalescent simulation with 10^5^ replicates under the two-phase mutation model (TPM), assuming a proportion of one-step mutations (ps) of 0.22 and a mean size of multi-step mutations (Δg) of 3.1. The significance of the deficiency in the M-ratio was tested using the Wilcoxon signed-rank test.

### 4.5. Clonal Diversity and Spatial Architecture

Clonal diversity was assessed using the function clonal_index implemented in the RClone package based on (1) clonal richness (R), is the proportion of unique genotypes present in a sample relative to the number of sampled ramets, described as R = (G − 1)/(N − 1), where G is the number of MLLs, and N is the number of ramets [118]; (2) Simpson’s diversity index (D), which estimates clonal heterogeneity and is defined as probability that two randomly selected individuals belong to the same MLL [119], and finally (3) Pielou index (J), which describes clonal architecture by assessing how evenly ramets are distributed among different genets [120]. To characterise the genet size (N_R_, the number of ramets belonging to each genet) and the distribution of clones within each study site, we used the same package.

RClone was also used to describe the spatial components of clonal growth across study sites. First, we estimated the spatial aggregation index (A_C_) at both the site and sex levels using the agg_index implemented in RClone. This metric estimates the relative probability that ramet pairs that are nearest neighbours belong to the same MLL compared to the average probability for all ramet pairs [73]. Values for A_C_ range between 0, when the probability between nearest neighbours does not differ from the global one, indicating that clonal replicates are dispersed, to 1 when all the nearest neighbours belong to the same MLL, indicating spatial aggregation. The significance of Ac was tested with 10 000 permutations. To determine the level of intermingling between genets, we calculated the clonal dominance index (D_C_) following the recommendation of Ohsako [4]. This metric describes the level of ramet aggregation within the range of a single clone. D_C_ was calculated separately for each sex and for each genet represented by at least three ramets as D_C_ = (NR − 1)/(NT − 1), where NR is the number of ramets belonging to the given MLL and NT is the total number of ramets (including those from other MLLs) present within its spatial range of the genet (GA). The genet range was defined as the areas occupied by all ramets of the target genet, calculated in the R environment as a convex hull. D_C_ equals 1 indicates that the genet area is exclusively occupied by ramets of the given MLL, indicating a highly compact, phalanx-type clonal architecture. Lower D_C_ values indicate greater intermingling of clones and a more diffuse clonal structure.

Further, to describe the scaling of the partitioning of the ramets among MLG size classes, we used the Pareto index. A cumulative function of the Pareto distribution to the data was fitted following Arnaud-Haond et al. [73] using the Pareto_index function. The regression slope (b_P_) was obtained from a double logarithmic linear regression of reverse cumulative frequency of genets containing *X* or more ramets (*N*_≥*X*_) against the number of sampled ramets belonging to a genet (*X*), and *R*^2^ represents the square of the correlation coefficient (coefficient of determination) between *N*_≥*X*_ and *X*. A skewed regression slope reflects a clonal distribution dominated by a few large clonal lineages alongside many smaller ones (a low β value). Larger values of this index indicate a more even distribution of ramets within clonal lineages. Also, for clones with at least three ramets, we calculate the mean and maximum distance among the nearest ramets of the same clone (d_near_) as a measure of size and spread of clonal patches for both sexes within study sites using QGIS.

Finally, we applied the Thomas double-cluster process [121] to illustrate spatial dependence in the occurrence of sexes within study sites. For each stand, the coordinates of all ramets were converted from geographic coordinates (WGS84) to projected metric coordinates (ETRS89/Poland CS92). Separate spatial point pattern objects were created for male and female individuals using the ppp function from the spatstat.geom package, v.3.3-2 [122]. Model fitting was conducted at the small cluster level using the kppm function from the spatstat.model package, v.3.7-2 [122], with the pair correlation function g(r) being the normalized neighbourhood density function calculated empirically with the function pcf and compared to theoretical expectations computed with the pcfmodel function. The g(r) represents the expected density of points (ramets) of an average point of the pattern at distance *r*, divided by the mean intensity (λ) of the point in the stand. The value of g(r) = 1 indicates a random pattern, while g(r) > 1 reflects spatial aggregation with a density of points higher than expected under a random pattern. To characterise the distribution of sexes within each study site, we used the following fitted model parameters: the mean number of ramets per cluster (μ), the cluster scale parameter (σ), and the density of clusters per m^2^ (ρ). Small clusters were designated using the density-based clustering algorithm from the dbscan package, v.1.2.6 [123], with the neighbourhood radius set to twice the estimated σ. Centroids of small clusters were calculated as the mean ramet coordinates. Model performance was further assessed through 199 Monte Carlo simulations of the fitted Thomas process. For each simulated point pattern, the function g(r) was recalculated, and the 5th and 95th percentiles were extracted to construct the simulation envelope. The observed g(r), model expectations, and simulation envelopes were plotted to assess the fit and detect potential deviations from the model assumptions. At the large cluster level, small clusters were aggregated based on distances between centroids using a threshold derived from the maximum of g(r) (> 1.05), indicating aggregation at a larger spatial scale. Large clusters were identified as connected components of an undirected graph constructed using the igraph package, v.2.0.3 [124], and their centroids were calculated as the mean coordinates of constituent small clusters. The resulting cluster hierarchy was visualised using ggplot2, v.4.0.0 [125].

### 4.6. Spatial Genetic Structure

To assess the effect of clonality and dioecy on SGS, we applied spatial autocorrelation analyses using SPAGeDi v.1.5 [126]. Analyses were conducted separately for males and females, and separately at both the genet and ramet levels. Average multilocus kinship coefficients (F_ij_) were estimated according to Nason’s F [127] for the following 15 distance classes: 25, 50, 100, 150, 200, 250, 300, 350, 400, 450, 500, 600, 700, 800, 1000 m. The distance classes were specified according to the recommendation of Hardy and Vekemans [126,128], such that the proportion of pairwise comparisons within a given spatial interval (% partic) exceeded 50%, and the coefficient of variation (CV partic) of the number of occurrences of each individual was ≤1. To visualise SGS, average F_ij_ were regressed against the natural logarithm of the distance between individuals, ln(d_ij_), which provides an estimate of the extent of SGS by obtaining the regression slope, b_F_. The significance of SGS was evaluated by calculating 95% confidence intervals for multilocus F_ij_ in each distance class as well as for b_F_ using 2 × 10^4^ permutations. The SGS intensity was evaluated based on the *Sp* statistic developed by Vekemans and Hardy [3], according to the formula: *Sp* = −b_F_/(1 − F_(1)_, where F_(1)_ refers to the average value of F_ij_ among individuals in the first distance class.

Wright’s neighbourhood size (Nb) and the mean-squared parent-offspring distance (σ) were estimated as indicators of gene dispersal and local genetic connectivity in the studied populations [3,126]. Nb was calculated from the estimated regression slope b_F_ as Nb = −(1 − F(1))/b_F_, and σ was subsequently inferred using the iterative procedure implemented in SPAGeDi, assuming a two-dimensional population at drift–dispersal equilibrium. This approach requires an estimate of effective population density (D), defined here as the density of sampled individuals (ramets) per unit area (ha), calculated based on the spatial extent of each study site (10.55 ha for RLP and 2.49 ha for DF). While using ramet density serves as a conservative proxy for effective density [129], we emphasize that Nb estimates remain robust as they are derived directly from the regression slope and are independent of the density parameter D. Starting from the global regression slope, σ and Nb were recalculated iteratively by restricting the regression to distances between σ and Xσ, where X defines the upper limit of the distance range. The procedure was repeated until σ converged, and estimates were retained only when convergence was achieved [128].

### 4.7. Population Genetic Substructure

To examine possible substructuring of the studied population, the Bayesian clustering approach implemented in Structure 2.3.4 [130] was applied. We first assessed overall genetic substructure by analysing all MLL within the population without any *a priori* classification based on sex. Subsequently, we conducted separate analyses of male and female MLL to investigate potential sex-specific patterns. The admixture and correlated allele frequencies models were applied. Before the analyses, we determined the number of genetic groups (K) by running simulations for K = 5, 10, and 15, selecting the optimal K using Evanno’s ΔK method [131], and spatially aggregating individuals of the same sex before conducting the final clustering estimations. In the final STRUCTURE analyses, ten independent runs were performed for each K, ranging from 1 to 10, to estimate individual admixture proportions using a burn-in period of 5 × 10^4^ steps followed by 5 × 10^5^ MCMC iterations. Structure Selector [132] was used to select the best-K value based on Evanno’s ΔK method, and to summarise and visualise the replicate runs with the integrate CLUMPACK program [133]. To visualise the genetic substructure at the population and sexes level, we spatially visualised it by interpolating mean membership coefficient values across the landscape using Inverse Distance Weighting (IDW) in QGIS.

## 5. Conclusions

The efforts are increasingly focused on developing evidence-based guidelines for riparian conservation, restoration, and management, including tools to assess ecological conditions and maintain the genetic resources of riparian trees [50,134,135,136]. A better understanding of how dynamic physical processes on the floodplain influence sexual and asexual recruitment of trees would ultimately improve the effectiveness of such strategies, and our study addresses these issues. Despite these challenges, only a few riparian trees have been studied in the context of genetic resource management. Among them, extensive research has focused on *Populus nigra,* providing a background for breeding, management and restoration programs [75,137]. Recently, attention has also been given to *Fraxinus excelsior* [138,139,140] and *Ulmus* species [141,142]. Given the recent prioritisation of genetic conservation in European riparian ecosystems [50], accurately assessing genetic diversity and the ecological processes shaping the gene dispersal of white poplar is crucial for guiding effective restoration efforts.

Our study demonstrated that dioecy and clonality can jointly strengthen SGS in a long-lived tree species, and that local habitat modulates the expression of these patterns. The observed female-biased SGS and sex-specific clonal architecture provide a more nuanced understanding of previously reported spatial dependencies in dioecious tree species [2,9,12,27]. In our study system, the dominance of vegetative reproduction and the detected bottleneck indicate an ongoing erosion of genetic resources in heavily modified riparian zones. While the long lifespan of clones (and trees in general) may partially slow the loss of genetic variation, it cannot replace the role of regular sexual recruitment in maintaining long-term adaptive potential. Furthermore, our findings demonstrate that pronounced spatial patterns should be considered in practical management to ensure the long-term persistence of white poplar populations under anthropogenic pressure and climate change.

Riparian zones are highly dynamic and complex systems that depend on flood regimes, which, in turn, influence clonal turnover and the balance between sexual and asexual reproduction, thereby shaping the establishment of the SGS patterns. Conservation and restoration efforts should therefore account for these processes and prioritise maintaining or restoring natural levels and patterns of gene flow, genetic diversity and opportunities for sexual recruitment. Accordingly, implementing long-term genetic monitoring should be considered a key priority for clonal tree species [50,143]. Incorporating knowledge about fine-scale SGS and sex-specific clonal aggregation into management planning can help define appropriate spatial scales for seed collection, planting schemes and thinning operations, thereby reducing the risk of inbreeding and the loss of genetic diversity in fragmented riparian stands. Future studies should integrate genetic and epigenetic analyses with direct estimates of gene flow and characterisation of microhabitat mosaics. Such integrative approaches will provide a deeper understanding of the environmental factors driving sexual and clonal regeneration, ultimately supporting management strategies aimed at preserving genetic variation and the adaptive potential of the species under climate change.

## Figures and Tables

**Table 1:** Genetic diversity parameters estimated at the genet level for the studied white poplar stands and the sex level, with the results of the M-ratio test under the two-phase model (TPM).

Stand		A	N_A_	H_O_	H_E_	A_P_	A_R_	F_IS_	Null	F_IS_Null	MR	MReq	*p*-Value
**RLP**	all	46	2.706	0.462	0.499	9	2.706	0.055	0.067	0.002	0.633	0.942	0.0009
**RLP**	female	46	2.706	0.450	0.478	0	2.706	0.042	0.063	0.003	0.655	0.936	0.0009
**RLP**	male	46	2.706	0.477	0.505	0	2.706	0.033	0.061	0.006	0.655	0.935	0.0008
**DF**	all	42	2.471	0.346	0.402	5	2.471	0.205	0.077	0.004	0.675	0.938	0.0021
**DF**	female	42	2.471	0.349	0.415	0	2.471	0.210	0.083	0.007	0.675	0.933	0.0021
**DF**	male	42	2.471	0.343	0.387	0	2.471	0.197	0.064	0.015	0.675	0.936	0.0021
***Populus alba***		44	2.589	0.404	0.451	7	2.589	0.13	0.072	0.003	-	-	-

RLP—Rogalin Landscape Park, DF—Dębina Forest, A—number of observed alleles, N_A_—average number of alleles, H_O_—observed proportion of heterozygotes, H_E_—expected proportion of heterozygotes, A_P_—private alleles, A_R_—allelic richness, F_IS_—inbreeding coefficient (F_IS_ = 1 − H_O_/H_E_), Null—null alleles, F_IS_Null—inbreeding coefficient with correction for null alleles (homozygosity excess result from presence of null alleles), MR—mean M-ratio, MReq—M-ratio generated under mutation-drift equilibrium, *p*-value—probability of significant test for the deficiency in M-ratio based on Wilcoxon signed-ranks test.

**Table 2:** Clonality and clonal structure for the studied white poplar stands.

Stand	Sex	Singl.	Clone	LCS	AIC	Ramet	d_near_min	d_near_max	d_near_mean	MLG	MLL	R	D	J	A_c_
**RLP**	all	96	78	45	5.19	405	0.20	236.75	16.59	174	174	0.346	0.976	0.864	0.171
**RLP**	female	61	36	44	5.24	209	0.41	236.75	21.44	97	97	0.357	0.952	0.828	0.233
**RLP**	male	35	42	45	5.19	196	0.20	53.28	11.55	77	77	0.330	0.951	0.862	0.028
**DF**	all	77	76	43	6.45	490	0.47	336.51	18.09	153	153	0.269	0.978	0.866	0.528
**DF**	female	39	35	43	6.00	250	0.47	336.51	16.05	74	74	0.263	0.949	0.823	0.639
**DF**	male	38	41	39	6.49	240	0.82	205.64	16.83	79	79	0.272	0.963	0.860	0.458

RLP—Rogalin Landscape Park, DF—Dębina Forest, LCS—largest clone size, AIC—average number of ramets per clone, d_near_max—maximum distance to the nearest ramet of the same clone (m), d_near_mean—average distance to the nearest ramet of the same clone (m), d_near_min—minimum distance to the nearest ramet of the same clone (m), MLG—multilocus genotypes, MLL—multilocus lineages, R—clonal richness, D—Simpson’s diversity index, J—Pielou index, A_C_—aggregation index, Singl.—Singleton.

**Table 3:** Parameters of the Thomas double-cluster process and clonal architecture described by the clonal dominance index (D_C)_, calculated for the studied white poplar stands.

Stand	Level	N	Large Clusters				Small Clusters				D_C_
			μ_1_	2*σ*_1_	ρ_1_	N Cluster	μ_2_	2*σ*_2_	ρ_2_	N Cluster	
**RLP**	all	501									0.708
	male	231	~38.5	54.049	0.1667	6	16.5	28.018	0.0714	14	0.796
	female	270	~34	30.485	0.125	8	19.28	28.209	0.0714	14	0.721
**DF**	all	567									0.651
	male	279	~48	31.028	0.17	6	14.4	17.583	0.05	20	0.719
	female	288	~35	33.591	0.125	8	16.4	25.114	0.0588	17	0.659

RLP—Rogalin Landscape Park, DF—Dębina Forest, N—number of individuals, μ—average number of individuals per cluster, 2σ—average diameter of cluster [m], ρ—density of clusters [number per m^2^], N cluster—number of clusters, Dc—Clonal dominance index.

**Table 4:** Spatial genetic structure parameters estimated at the genet and ramet levels for the studied white poplar stands and sexes.

Stand	Level	F_ij (25m)_	b_F_	Sp	Nb	σ
RLP	genets	0.170 (0.004) *	−0.038 (0.0008) *	0.045	29.795	0.022
	ramets	0.365 (0.0019) *	−0.074 (0.0003) *	0.116		
	female genets	0.159 (0.0072) *	−0.027 (0.0011) *	0.033	NC	NC
	female ramets	0.374 (0.0041) *	−0.081 (0.0007) *	0.129		
	male genets	0.236 (0.0094) *	−0.055 (0.0017) *	0.072	14.900	0.016
	male ramets	0.322 (0.0041) *	−0.078 (0.0007) *	0.115		
DF	genets	0.106 (0.0048) *	−0.042 (0.0014) *	0.047	21.020	0.009
	ramets	0.190 (0.0014) *	−0.069 (0.0004) *	0.085		
	female genets	0.188 (0.0109) *	−0.065 (0.0031) *	0.080	7.747	0.005
	female ramets	0.354 (0.0036) *	−0.115 (0.0009) *	0.177		
	male genets	0.120 (0.0092) *	−0.038 (0.0027) *	0.043	28.717	0.010
	male ramets	0.171 (0.0025) *	−0.055 (0.0006) *	0.066		

RLP—Rogalin Landscape Park, DF—Dębina Forest, F_ij (25 m)_—average kinship coefficient between individuals from the first distance class, given with SD, b_F_—the regression slope of F_ij_ on log spatial distance given with SD, Sp—intensity of SGS, Nb—the neighbourhood size, σ—gene dispersal distance (km), * significant at 0.05, NC—no convergence for gene dispersal estimate.

## Data Availability

The original contributions presented in this study are included in the article/Supplementary Materials. Further inquiries can be directed to the corresponding author.

## Supplementary Materials

The following supporting information can be downloaded at: https://www.mdpi.com/article/10.3390/ijms27208955/s1.

## References

[1] Epperson B.K. (2007). Plant Dispersal, Neighbourhood Size and Isolation by Distance. Mol. Ecol..

[2] Vallejo-Marín M., Dorken M.E., Barrett S.C.H. (2010). The Ecological and Evolutionary Consequences of Clonality for Plant Mating. Annu. Rev. Ecol. Evol. Syst..

[3] Vekemans X., Hardy O.J. (2004). New Insights from Fine-Scale Spatial Genetic Structure Analyses in Plant Populations. Mol. Ecol..

[4] Ohsako T. (2010). Clonal and Spatial Genetic Structure within Populations of a Coastal Plant, *Carex kobomugi* (Cyperaceae). Am. J. Bot..

[5] Dering M., Chybicki I.J., Rączka G. (2015). Clonality as a Driver of Spatial Genetic Structure in Populations of Clonal Tree Species. J. Plant Res..

[6] Holt R., Kwok A., Dorken M.E. (2020). Increased Spatial-Genetic Structure in a Population of the Clonal Aquatic Plant *Sagittaria latifolia* (Alismataceae) Following Disturbance. Heredity.

[7] Reusch T.B.H., Hukriede W., Stam W.T., Olsen J.L. (1999). Differentiating between Clonal Growth and Limited Gene Flow Using Spatial Autocorrelation of Microsatellites. Heredity.

[8] Chenault N., Arnaud-Haond S., Juteau M., Valade R., Almeida J.-L., Villar M., Bastien C., Dowkiw A. (2011). SSR-Based Analysis of Clonality, Spatial Genetic Structure and Introgression from the Lombardy Poplar into a Natural Population of *Populus nigra* L. along the Loire River. Tree Genet. Genomes.

[9] Dering M., Rączka G., Szmyt J. (2016). Sex-Specific Pattern of Spatial Genetic Structure in Dioecious and Clonal Tree Species, *Populus alba* L.. Tree Genet. Genomes.

[10] Reisch C., Schurm S., Poschlod P. (2007). Spatial Genetic Structure and Clonal Diversity in an Alpine Population of *Salix herbacea* (Salicaceae). Ann. Bot..

[11] Castiglione S., Cicatelli A., Lupi R., Patrignani G., Fossati T., Brundu G., Sabatti M., Loo M., Lexer C. (2010). Genetic Structure and Introgression in Riparian Populations of *Populus alba* L.. Plant Biosyst..

[12] Nazareno A.G., Alzate-Marin A.L., Pereira R.A.S. (2013). Dioecy, More than Monoecy, Affects Plant Spatial Genetic Structure: The Case Study of *Ficus*. Ecol. Evol..

[13] Chybicki I.J., Dering M., Iszkuło G., Meyza K., Suszka J. (2016). Relative Strength of Fine-Scale Spatial Genetic Structure in Paternally vs. Biparentally Inherited DNA in a Dioecious Plant Depends on Both Sex Proportions and Pollen-to-Seed Dispersal Ratio. Heredity.

[14] Carvalho C.d.S., da Costa L.E.N., Leal B.S.S., Silva K.R., Valentin-Silva A., Costa A.C.G., Tyski L., dos Santos F.M.G., Watanabe M.T.C. (2024). Mating System, Morphological and Genetic Evidence Endorse Clonality as an Essential Reproductive Mode in *Daphnopsis filipedunculata* (Thymelaeaceae), a Dioecious and Endemic Species from the Amazon. AoB Plants.

[15] Bisbing S.M., Urza A.K., Buma B.J., Cooper D.J., Matocq M., Angert A.L. (2021). Can Long-Lived Species Keep Pace with Climate Change? Evidence of Local Persistence Potential in a Widespread Conifer. Divers. Distrib..

[16] Cruz-Salazar B., Flores-Manzanero A., Navarro-Noya Y., Rico Y. (2024). Spatial Genetic Structure of Two Conifers in a Highly Human-Modified Landscape of Central Mexico. Tree Genet. Genomes.

[17] Brzyski J.R., Stieha C.R., Nicholas McLetchie D. (2018). The Impact of Asexual and Sexual Reproduction in Spatial Genetic Structure within and between Populations of the Dioecious Plant *Marchantia inflexa* (Marchantiaceae). Ann. Bot..

[18] Pfeiffer T., Schmidt L., Roschanski A.M., Schnittler M. (2019). A Battle of the Sexes? Sex Ratio and Performance at Small Scales in Dioecious *Mercurialis perennis*. Acta Acol..

[19] Charpentier A. (2001). Consequences of Clonal Growth for Plant Mating. Evol. Ecol..

[20] Vange V. (2002). Breeding System and Inbreeding Depression in the Clonal Plant Species *Knautia arvensis* (Dipsacaceae): Implications for Survival in Abandoned Grassland. Biol. Conserv..

[21] Binks R.M., Millar M.A., Byrne M. (2015). Contrasting Patterns of Clonality and Fine-Scale Genetic Structure in Two Rare Sedges with Differing Geographic Distributions. Heredity.

[22] Xu N.-N., Jiang K., Biswas S.R., Tong X., Wang R., Chen X.-Y. (2019). Clone Configuration and Spatial Genetic Structure of Two *Halophila ovalis* Populations With Contrasting Internode Lengths. Front. Ecol. Evol..

[23] Van Loo M., Joseph J.A., Heinze B., Fay M.F., Lexer C. (2008). Clonality and Spatial Genetic Structure in *Populus x canescens* and Its Sympatric Backcross Parent *P. alba* in a Central European Hybrid Zone. New Phytol..

[24] Hultine K.R., Bush S.E., West A.G., Ehleringer J.R. (2007). Population Structure, Physiology and Ecohydrological Impacts of Dioecious Riparian Tree Species of Western North America. Oecologia.

[25] Scheuerell R.P., LeRoy C.J. (2023). Plant Sex Influences on Riparian Communities and Ecosystems. Ecol. Evol..

[26] Schroeder J., Tran H., Dick C. (2014). Fine Scale Spatial Genetic Structure in *Pouteria reticulata* (Engl.) Eyma (Sapotaceae), a Dioecious, Vertebrate Dispersed Tropical Rain Forest Tree Species. Glob. Ecol. Conserv..

[27] Heilbuth J.C., Ilves K.L., Otto S.P. (2001). The Consequences of Dioecy for Seed Dispersal: Modeling the Seed-Shadow Handicap. Evolution.

[28] Petzold A., Pfeiffer T., Jansen F., Eusemann P., Schnittler M. (2013). Sex Ratios and Clonal Growth in Dioecious *Populus euphratica* Oliv., Xinjiang Prov., Western China. Trees.

[29] Timerman D., Barrett S.C.H. (2019). The Spatial Ecology of Sex Ratios in a Dioecious Plant: Relations between Ramet and Genet Sex Ratios. J. Ecol..

[30] Brzyski J.R., Taylor W., McLetchie D.N. (2014). Reproductive Allocation between the Sexes, across Natural and Novel Habitats, and Its Impact on Genetic Diversity. Evol. Ecol..

[31] Van Drunen W.E., van Kleunen M., Dorken M.E. (2015). Consequences of Clonality for Sexual Fitness: Clonal Expansion Enhances Fitness under Spatially Restricted Dispersal. Proc. Natl. Acad. Sci. USA.

[32] Liu M., Korpelainen H., Li C. (2021). Sexual Differences and Sex Ratios of Dioecious Plants under Stressful Environments. J. Plant Ecol..

[33] Wan Y., Ding N., Tian T., Sun K., Fan B. (2025). Sex-Specific Differences in the Clonality of *Hippophae tibetana* at Different Altitudes in Alpine Meadows of the Eastern Qinghai–Tibet Plateau. Forests.

[34] Caudullo G., de Rigo D. (2016). Populus Alba in Europe: Distribution, Habitat, Usage and Threats. European Atlas of Forest Tree Species.

[35] Havrdová A., Douda J., Doudová J. (2023). Threats, Biodiversity Drivers and Restoration in Temperate Floodplain Forests Related to Spatial Scales. Sci. Total Environ..

[36] Hughes F.M.R., Rood S.B. (2003). Allocation of River Flows for Restoration of Floodplain Forest Ecosystems: A Review of Approaches and Their Applicability in Europe. Environ. Manag..

[37] Palancean I., Alba N., Sabatti M., De Vries S.M.G. (2018). EUFORGEN Technical Guidelines for Genetic Conservation and Use of European White Poplar (*Populus alba*). https://www.euforgen.org/fileadmin/templates/euforgen.org/upload/Publications/Technical_guidelines/Technical_guidelines_Populus_alba.pdf.

[38] Hughes F.M.R., del Tánago M.G., Mountford J.O., Stanturf J., Madsen P., Lamb D. (2012). Restoring Floodplain Forests in Europe. A Goal-Oriented Approach to Forest Landscape Restoration.

[39] Mansourian S., Doncheva N., Valchev K., Vallauri D. (2019). Lessons Learnt from 20 Years of Floodplain Forest Restoration: The Lower Danube Landscape.

[40] Portela A.P., Vieira C., Carvalho-Santos C., Gonçalves J., Durance I., Honrado J. (2021). Regional Planning of River Protection and Restoration to Promote Ecosystem Services and Nature Conservation. Landsc. Urban Plan..

[41] Tierney D.A., Ahrens C., Rymer P., Auld T.D. (2020). The Interaction of Clonality, Breeding System and Genomics for a Highly Threatened Plant Species and the Management Implications. Biodivers. Conserv..

[42] Fossati T., Patrignani G., Zapelli I., Sabatti M., Sala F., Castiglione S. (2004). Development of Molecular Markers to Assess the Level of Introgression of *Populus tremula* into *P. alba* Natural Populations. Plant Breed..

[43] Brundu G., Lupi R., Zapelli I., Fossati T., Patrignani G., Camarda I., Sala F., Castiglione S. (2008). The Origin of Clonal Diversity and Structure of *Populus alba* in Sardinia: Evidence from Nuclear and Plastid Microsatellite Markers. Ann. Bot..

[44] Santos del Blanco L., de-Lucas A., González-Martínez S., Sierra-de-Grado R., Hidalgo E. (2012). Extensive Clonal Assemblies in *Populus alba* and *Populus x canescens* from the Iberian Peninsula. Tree Genet. Genomes.

[45] Zeng Y.-F., Zhang J., Duan A.G., Abuduhamiti B. (2016). Genetic Structure of *Populus* Hybrid Zone along the Irtysh River Provides Insight into Plastid-Nuclear Incompatibility. Sci. Rep..

[46] Tiwari B., Singh J., Sharma S., Sharma Y., Sharma K. (2023). Genetic Diversity of Populus Alba L in Kinnaur, Himachal Pradesh, India. Int. J. Farm Sci..

[47] Uzan Eken B., Kirdök E., Velioğlu E., Çiftçi Y.Ö. (2024). Genetic Variation in White Poplar (*Populus alba* L.) Populations as Characterized by SSR Markers. Turk. J. Bot..

[48] Tulik M., Grochowina A., Jura-Morawiec J., Bijak S. (2020). Groundwater Level Fluctuations Affect the Mortality of Black Alder (*Alnus glutinosa* Gaertn.). Forests.

[49] Hultine K.R., Bush S.E., Ward J.K., Dawson T.E. (2018). Does Sexual Dimorphism Predispose Dioecious Riparian Trees to Sex Ratio Imbalances under Climate Change?. Oecologia.

[50] Alimpić F., Milovanović J., Pielech R., Hinkov G., Jansson R., Dufour S., Beza M., Bilir N., del Blanco L.S., Božič G. (2022). The Status and Role of Genetic Diversity of Trees for the Conservation and Management of Riparian Ecosystems: A European Experts’ Perspective. J. Appl. Ecol..

[51] Namroud M.-C., Park A., Tremblay F., Bergeron Y. (2005). Clonal and Spatial Genetic Structures of Aspen (*Populus tremuloides* Michx.). Mol. Ecol..

[52] Hoebee S.E., Menn C., Rotach P., Finkeldey R., Holderegger R. (2006). Spatial Genetic Structure of *Sorbus Torminalis*: The Extent of Clonal Reproduction in Natural Stands of a Rare Tree Species with a Scattered Distribution. For. Ecol. Manag..

[53] Michel J.T., Evans J.P., Fox S.J., Morris A.B. (2026). Long-Term Demography and Spatial Genetic Structure Reveal Mechanisms of *Sassafras albidum* Population Persistence through Clonality. Am. J. Bot..

[54] Silvertown J. (2008). The Evolutionary Maintenance of Sexual Reproduction: Evidence from the Ecological Distribution of Asexual Reproduction in Clonal Plants. Int. J. Plant Sci..

[55] Eriksson O. (1989). Seedling Dynamics and Life Histories in Clonal Plants. Oikos.

[56] Honnay O., Bossuyt B. (2005). Prolonged Clonal Growth: Escape Route or Route to Extinction?. Oikos.

[57] Jolivet C., Degen B. (2011). Spatial Genetic Structure in Wild Cherry (*Prunus avium* L.): II. Effect of Density and Clonal Propagation on Spatial Genetic Structure Based on Simulation Studies. Tree Genet. Genomes.

[58] Kramp K., Schmitt T., Lang P., Jeschke M., Schäfer P., Kulanek D., Zhang X., Yu R., Thomas F.M. (2018). Clones or No Clones: Genetic Structure of Riparian *Populus euphratica* Forests in Central Asia. J. Arid Land.

[59] Jankowska-Wróblewska S., Kozioł C., Ulaszewski B., Burczyk J. (2026). Demographic and Environmental Determinants of Clonality in a Self-Incompatible Scattered Tree Species, *Sorbus torminalis* (L.) Crantz. Forestry.

[60] Karrenberg S., Suter M. (2003). Phenotypic Trade-Offs in the Sexual Reproduction of Salicaceae from Flood Plains. Am. J. Bot..

[61] Suszka B. (1983). Generatywne Rozmnażanie Topoli. https://www.rcin.org.pl/id/Content/164314/PDF/KOR001_154309_2006_Rozmnazanie-generaty.pdf.

[62] Mahoney J.M., Rood S.B. (1998). Streamflow Requirements for Cottonwood Seedling Recruitment—An Integrative Model. Wetlands.

[63] González E., Comín F.A., Muller E. (2010). Seed Dispersal, Germination and Early Seedling Establishment of *Populus alba* L. under Simulated Water Table Declines in Different Substrates. Trees.

[64] Edwards P.J. (2024). Ecological Traits of Saliceae and the Species Replacing Them on the Active Floodplain. River Res. Appl..

[65] Ally D., Ritland K., Otto S.P. (2010). Aging in a Long-Lived Clonal Tree. PLoS Biol..

[66] Van Drunen W.E., Dorken M.E. (2014). Wind Pollination, Clonality, and the Evolutionary Maintenance of Spatial Segregation of the Sexes. Evol. Ecol..

[67] Barrett S.C.H. (2015). Influences of Clonality on Plant Sexual Reproduction. Proc. Natl. Acad. Sci. USA.

[68] de Witte L.C., Stöcklin J. (2010). Longevity of Clonal Plants: Why It Matters and How to Measure It. Ann. Bot..

[69] Ally D., Ritland K., Otto S.P. (2008). Can Clone Size Serve as a Proxy for Clone Age? An Exploration Using Microsatellite Divergence in *Populus tremuloides*. Mol. Ecol..

[70] Barsoum N., Muller E., Skot L. (2004). Variations in Levels of Clonality among *Populus nigra* L. Stands of Different Ages. Evol. Ecol..

[71] Cristobal D., Martínez-Zurimendi P., Villamediana I., Ciriza J., Villar J., Nanos N., Sierra-de-Grado R. (2014). Clonal Structure and Dynamics of Peripheral *Populus tremula* L. Populations. iForest—Biogeosci. For..

[72] Robak D., Lewandowski A., Żukowska W. (2024). Genetic Divergence in the Natural Regeneration of Black Poplar Along the Vistula River in Poland. Aquat. Conserv. Mar. Freshw. Ecosyst..

[73] Arnaud-Haond S., Duarte C., Alberto F., Serrao E. (2008). Standardizing Method to Address Clonality in Population Studies. Mol. Ecol..

[74] Wójkiewicz B., Lewandowski A., Żukowska W.B., Litkowiec M., Wachowiak W. (2021). Low Effective Population Size and High Spatial Genetic Structure of Black Poplar Populations from the Oder Valley in Poland. Ann. For. Sci..

[75] Żukowska W., Lewandowski A. (2025). Genetic Structure and Divergence of Marginal Populations of Black Poplar (*Populus nigra* L.) in Poland. Sci. Rep..

[76] Bond W.J., Midgley J.J. (2001). Ecology of Sprouting in Woody Plants: The Persistence Niche. Trends Ecol. Evol..

[77] Barsoum N. (2001). Relative Contributions of Sexual and Asexual Regeneration Strategies in *Populus nigra* and *Salix alba* during the First Years of Establishment on a Braided Gravel Bed River. Evol. Ecol..

[78] Fischer S., Greet J., Walsh C.J., Catford J.A. (2021). Flood Disturbance Affects Morphology and Reproduction of Woody Riparian Plants. Sci. Rep..

[79] Bona A., Brzeziński D., Jadwiszczak K.A. (2022). Genetic Diversity and Fine-Scale Spatial Genetic Structure of the Endangered Shrub Birch (*Betula humilis* Schrk.) Populations in Protected and Unprotected Areas. Diversity.

[80] Sandurska E., Ulaszewski B., Meyza K., Sztupecka E., Burczyk J. (2024). Factors Determining Fine-Scale Spatial Genetic Structure within Coexisting Populations of Common Beech (*Fagus sylvatica* L.), Pedunculate Oak (*Quercus robur* L.), and Sessile Oak (*Q. petraea* (Matt.) Liebl.). Ann. For. Sci..

[81] Chung M.Y., Suh Y., López-Pujol J., Nason J.D., Chung M.G. (2005). Clonal and Fine-Scale Genetic Structure in Populations of a Restricted Korean Endemic, *Hosta jonesii* (Liliaceae) and the Implications for Conservation. Ann. Bot..

[82] Peng Y., Macek P., Macková J., Romoleroux K., Hensen I. (2015). Clonal Diversity and Fine-scale Genetic Structure in a High Andean Treeline Population. Biotropica.

[83] Nilsson C., Brown R.L., Jansson R., Merritt D.M. (2010). The Role of Hydrochory in Structuring Riparian and Wetland Vegetation. Biol. Rev..

[84] Mock K.E., Rowe C.A., Hooten M.B., Dewoody J., Hipkins V.D. (2008). Clonal Dynamics in Western North American Aspen (*Populus tremuloides*). Mol. Ecol..

[85] Loh R., Scarano F.R., Alves-Ferreira M., Salgueiro F. (2015). Clonality Strongly Affects the Spatial Genetic Structure of the Nurse Species *Aechmea nudicaulis* (L.) Griseb. (Bromeliaceae). Bot. J. Linn. Soc..

[86] Serna-Sánchez M.A., Karremans A.P., Bogarín D., Fuchs E.J. (2025). Contrasting Clonal and Population Genetic Structure in Two Endangered Costa Rican Vanilla Species of Commercial Interest. Sci. Rep..

[87] Oborny B., Cain M. (1997). Models of Spatial Spread and Foraging in Clonal Plant Species. The Ecology and Evolution of Clonal Plants.

[88] Klimešová J., Ottaviani G., Charles-Dominique T., Campetella G., Canullo R., Chelli S., Janovský Z., Lubbe F.C., Martínková J., Herben T. (2021). Incorporating Clonality into the Plant Ecology Research Agenda. Trends Plant Sci..

[89] Stuefer J.F. (1998). Two Types of Division of Labour in Clonal Plants: Benefits, Costs and Constraints. Perspect. Plant Ecol. Evol. Syst..

[90] Oborny B., Mony C., Herben T. (2012). From Virtual Plants to Real Communities: A Review of Modelling Clonal Growth. Ecol. Model..

[91] Bouteiller X.P., Bussolo M., Ségura R., Mariette S., Porté A.J. (2023). Considering Both Sexual and Clonal Reproduction Could Help Loosen the Conflict of Use over *Robinia pseudoacacia*. Dendrobiology.

[92] Imbert E., Lefèvre F. (2003). Dispersal and Gene Flow of *Populus nigra* (Salicaceae) along a Dynamic River System. J. Ecol..

[93] Pospíšková M., Šálková I. (2006). Population Structure and Parentage Analysis of Black Poplar along the Morava River. Can. J. For. Res..

[94] Liao D., Lei J., Wang Y., Bao Y., Zhang X., Wang J. (2025). Sex-Specific Responses of Sexual Reproduction, Clonal Reproduction, and Vegetative Growth to Environmental (Biotic and Abiotic) Factors in the Clonal Dioecious Plant *Acer barbinerve*. Plants.

[95] Fujitaka T., Sakai S. (2007). Sexual Dimorphism in Clonal Growth Forms and Ramet Distribution Patterns in *Rumex ccetosella* (Polygonaceae). Ecol. Res..

[96] Matsushita M., Takao M., Makita A. (2016). Sex-Different Response in Growth Traits to Resource Heterogeneity Explains Male-Biased Sex Ratio. Acta Acol..

[97] Iszkuło G., Jasińska A., Sobierajska K. (2011). Dendroecological Differences between *Taxus baccata* Males and Females in Comparison with Monoecious *Abies alba*. Dendrobiology.

[98] Bierzychudek P., Eckhart V. (1988). Spatial Segregation of the Sexes of Dioecious Plants. Am. Nat..

[99] Lexer C., Fay M.F., Joseph J.A., Nica M.-S., Heinze B. (2005). Barrier to Gene Flow between Two Ecologically Divergent Populus Species, *P. alba* (White Poplar) and *P. tremula* (European Aspen): The Role of Ecology and Life History in Gene Introgression. Mol. Ecol..

[100] Tylkowski T. (2010). Przekształcenia w składzie dendroflory w dolinie środkowej Warty. Acta Sci. Pol. Adm. Locorum.

[101] Broadhurst L.M., Lowe A., Coates D.J., Cunningham S.A., McDonald M., Vesk P.A., Yates C. (2008). Seed Supply for Broadscale Restoration: Maximizing Evolutionary Potential. Evol. Appl..

[102] Breed M.F., Stead M.G., Ottewell K.M., Gardner M.G., Lowe A.J. (2013). Which Provenance and Where? Seed Sourcing Strategies for Revegetation in a Changing Environment. Conserv. Genet..

[103] Frankham R., Ballou J.D., Eldridge M.D.B., Lacy R.C., Ralls K., Dudash M.R., Fenster C.B. (2011). Predicting the Probability of Outbreeding Depression. Conserv. Biol..

[104] Dumolin S., Demesure B., Petit R. (1995). Inheritance of Chloroplast and Mitochondrial Genomes in Pedunculate Oak Investigated with an Efficient PCR Method. TAG Theor. Appl. Genet. Theor. Angew. Genet..

[105] Tuskan G.A., Gunter L.E., Yang Z.K., Yin T., Sewell M.M., DiFazio S.P. (2004). Characterization of Microsatellites Revealed by Genomic Sequencing of *Populus trichocarpa*. Can. J. For. Res..

[106] Van Der Schoot J., Pospiskova M., Vosman N., Smulders M.J.M. (2000). Development and Characterization of Microsatellite Markers in Black Poplar (*Populus nigra* L). Theor. Appl. Genet..

[107] Igor Chybicki Raw2Gen. https://www.ukw.edu.pl/pracownicy/strona/igor_chybicki/software_ukw/.

[108] Bailleul D., Stoeckel S., Arnaud-Haond S. (2016). RClone: A Package to Identify multiLocus Clonal Lineages and Handle Clonal Datasets in R. Methods Ecol. Evol..

[109] Kamvar Z., Tabima J., Grünwald N. (2014). Poppr: An R Package for Genetic Analysis of Populations with Clonal, Partially Clonal, and/or Sexual Reproduction. PeerJ.

[110] Schnittler M., Eusemann P. (2010). Consequences of Genotyping Errors for Estimation of Clonality: A Case Study on *Populus euphratica* Oliv. (Salicaceae). Evol. Ecol..

[111] Young A.G., Hill J.H., Murray B.G., Peakall R. (2002). Breeding System, Genetic Diversity and Clonal Structure in the Sub-Alpine Forb *Rutidosis Leiolepis* F. Muell. (Asteraceae). Biol. Conserv..

[112] Rousset F. (2008). Genepop’007: A Complete Re-Implementation of the Genepop Software for Windows and Linux. Mol. Ecol. Resour..

[113] Chybicki I., Burczyk J. (2009). Simultaneous Estimation of Null Alleles and Inbreeding Coefficients. J. Hered..

[114] Goudet J. (2005). Hierfstat, a Package for r to Compute and Test Hierarchical F-Statistics. Mol. Ecol. Notes.

[115] Khan N. (2023). Introduction to Statistical Tests of Significance. Res. Rev. J. Stat..

[116] Garza J.C., Williamson E.G. (2001). Detection of Reduction in Population Size Using Data from Microsatellite Loci. Mol. Ecol..

[117] Cornuet J.M., Luikart G. (1996). Description and Power Analysis of Two Tests for Detecting Recent Population Bottlenecks from Allele Frequency Data. Genetics.

[118] Dorken M., Eckert C. (2001). Severely Reduced Sexual Reproduction in Northern Populations of a Clonal Plant, *Decodon verticillatus* (Lythraceae). J. Ecol..

[119] Pielou E.C. (1969). An Introduction to Mathematical Ecology.

[120] Pielou E.C. (1975). Ecological Diversity.

[121] Thomas M. (1949). A Generalization of Poisson’s binomial limit for use in ecology. Biometrika.

[122] Baddeley A., Rubak E., Turner R. (2015). Spatial Point Patterns: Methodology and Applications with R.

[123] Hahsler M., Piekenbrock M., Doran D. (2019). Dbscan: Fast Density-Based Clustering with R. J. Stat. Softw..

[124] Csárdi G., Nepusz T. (2006). The Igraph Software Package for Complex Network Research. https://r.igraph.org/authors.html.

[125] Wickham H. (2016). Data Analysis. ggplot2.

[126] Hardy O., Vekemans X. (2002). SPAGeDI: A Versatile Computer Program to Analyse Spatial Genetic Structure at the Individual or Population Levels. Mol. Ecol. Notes.

[127] Loiselle B.A., Sork V.L., Nason J., Graham C. (1995). Spatial Genetic Structure of a Tropical Understory Shrub, *Psychotria officinalis* (Rubiacea). Am. J. Bot..

[128] Hardy O.J., Vekemans X. (2015). SPAGeDi 1.5 Manual.

[129] Dodd R.S., Mayer W., Nettel A., Afzal-Rafii Z. (2013). Clonal Growth and Fine-Scale Genetic Structure in Tanoak (*Notholithocarpus densiflorus*: Fagaceae). J. Hered..

[130] Pritchard J., Stephens M., Donnelly P. (2000). Inference of Population Structure Using Multilocus Genotype Data. Genetics.

[131] Evanno G., Regnaut S., Goudet J. (2005). Detecting the Number of Clusters of Individuals Using the Software Structure: A Simulation Study. Mol. Ecol..

[132] Li Y., Liu J. (2018). StructureSelector: A Web-based Software to Select and Visualize the Optimal Number of Clusters Using Multiple Methods. Mol. Ecol. Resour..

[133] Kopelman N., Mayzel J., Jakobsson M., Rosenberg N., Mayrose I. (2015). CLUMPAK: A Program for Identifying Clustering Modes and Packaging Population Structure Inferences across K. Mol. Ecol. Resour..

[134] Urbanič G., Politti E., Rodríguez-González P.M., Payne R., Schook D., Alves M.H., Anđelković A., Bruno D., Chilikova-Lubomirova M., Di Lonardo S. (2022). Riparian Zones—From Policy Neglected to Policy Integrated. Front. Environ. Sci..

[135] Kuglerová L., Muotka T., Chellaiah D., Jyväsjärvi J., Richardson J.S. (2024). Protecting Our Streams by Defining Measurable Targets for Riparian Management in a Forestry Context. J. Appl. Ecol..

[136] Spitzer R., Kuglerová L., Löfroth T., Maher-Hasselquist E. (2025). Managing the Margins? Ungulate Forage Availability in Riparian Zones of Forest Streams and Ditches in Sweden. For. Ecol. Manag..

[137] Storme V., Vanden Broeck A., Ivens B., Halfmaerten D., Van Slycken J., Castiglione S., Grassi F., Fossati T., Cottrell J.E., Tabbener H.E. (2004). Ex-Situ Conservation of Black Poplar in Europe: Genetic Diversity in Nine Gene Bank Collections and Their Value for Nature Development. Theor. Appl. Genet..

[138] Ruņģis D., Korica A., Gailīte A., Pušpure I., Veinberga I. (2016). Analysis of the Genetic Diversity and Population Structure of Latvian Ash (*Fraxinus excelsior* L.) Stands Using Nuclear and Chloroplast SSR Markers. Proc. Latv. Acad. Sci. Sect. B Nat. Exact. Appl. Sci..

[139] Meger J., Kozioł C., Pałucka M., Burczyk J., Chybicki I.J. (2024). Genetic Resources of Common Ash (*Fraxinus excelsior* L.) in Poland. BMC Plant Biol..

[140] Nowakowska J., Słowik J., Pacia A., Tereba A., Marozau A., Borowik P., Oszako T. (2025). Interplay Between Genetic Diversity and Tree Vitality in Fraxinus Excelsior Populations Affected by Ash Dieback. Genes.

[141] Fuentes-Utrilla P., Valbuena-Carabaña M., Ennos R., Gil L. (2014). Population Clustering and Clonal Structure Evidence the Relict State of *Ulmus minor* Mill. in the Balearic Islands. Heredity.

[142] Collin E., Bozzano M. (2015). Implementing the Dynamic Conservation of Elm Genetic Resources in Europe: Case Studies and Perspectives. iForest—Biogeosci. For..

[143] Surmacz B., Rodríguez González P.M., Jansson R., Suchan T., Pielech R. (2025). Global-Scale Analysis Reveals Importance of Environment and Species Traits in Spatial Patterns of Riparian Plants’ Genetic Diversity. Glob. Ecol. Biogeogr..

