# Supplementary material for: Being Female Matters: Drivers of Spatial Genetic Structure in Clonal White Poplar
Source: Int J Mol Sci. 2026 Oct 9;27(20):8955. doi: 10.3390/ijms27208955 (PMC13704438; doi:10.3390/ijms27208955)
Supplement: Supplementary file 1 [file ijms-27-08955-s001.zip › Supplementary_file_1.pdf]

Table S1. Genetic diversity parameters estimated for each marker in both study sites.

General information						Rogalin Landcape Park					Dębina Forest				
**Locus**	**Primer sequence (forward. reverse)**	**SSR motif**	**Allelic range**	**Annealing temperature**	**Multiplex**	**A**	**H_O**	**H_E**	**F_{IS}**	**Null**	**A**	**H_O**	**H_E**	**F_{IS}**	**Null**
ORPM021	F: GGCTGCAGCACCAGAATAAT<br>R: TGCATCCAAAATTTTCCTCTTT	$(AG)_4$	206-216	57°C	2	2	0.149	0.158	0.054	0.013	2	0	0.178	1	0.199
ORPM028	F: GGATCGACTTCCAACCCATA<br>R: AATTCCCAGATGAAGGCTCA	$(AT)_7$	197-206	57°C	2	2	0.414	0.438	0.055	0.012	2	0.399	0.35	-0.14	0.005
ORPM030. ORPM30-2	F: ATGTCCACACCCAGATGACA	$(TC)_9$	212-234	48°C	2	4	0.494	0.706	0.3	0.116	4	0.51	0.703	0.275	0.107
	R: CCGGCTTCATTAAGAGTTGG					3	0.902	0.589	-0.53	0.001	3	0.778	0.561	-0.39	0.002
ORPM149	F: GTCTCTGCCACATGATCCAA<br>R: CCCGAAATGGATCAAACAAG	$(AT)_4…(CT)_4$	220-230	55°C	3	3	0.431	0.519	0.169	0.049	3	0.19	0.536	0.647	0.230
ORPM186	F: GGCTAGGAATACCCTGGAGAA<br>R: AAGCCATCTCGACTATACACCA	$(TTTA)_5$	240-250	55°C	3	2	0.293	0.346	0.152	0.027	1	0	0	0	0
ORPM190	F: CCCTGGTTTTCTCTTCTTGG<br>R: CCAGATTGGACTTGGGATTC	$(TG)_7$	180-204	55°C	3	2	0.92	0.497	-0.85	0.001	1	0	0	0	0
ORPM214	F: TTTTCACAGCCTCGAAGGA<br>R: TGGAAGACCCGAACTTTTTC	$(TC)_{11}$	160-170	55°C	3	2	0.431	0.339	-0.27	0.002	1	0	0	0	0
ORPM312. ORPM312-2	F: GTGGGGATCAATCCAAAAGA	$(CCT)_6$	220-260	48°C	2	3	0.282	0.536	0.475	0.173	3	0.203	0.376	0.461	0.153
	R: CCCATATCAAACCATTTGAAAAA					3	0.466	0.668	0.303	0.116	3	0.595	0.625	0.048	0.023
PMGC2852	F: ATAATCTCCCTAGCTTAATTCC<br>R: GAATAACATGGATAATGTGTTTG	(GA)	93-113	48°C	4	3	0.443	0.592	0.253	0.093	3	0.601	0.655	0.083	0.037
WPMS05	F: TTCTTTTTCAACTGCCTAACTT<br>R: TGATCCAATAACAGACAGAACA	$(GT)_{27}$	270–308	48°C	1	3	0.356	0.486	0.267	0.101	3	0.373	0.471	0.209	0.068
WPMS06	F: GTATAACGATGACCCCACGAAGAC<br>R: TATAAATAAAGGCATGACCAGACA	$(GT)_{24}$	182–226	48°C	1	2	0.167	0.326	0.489	0.134	2	0	0.178	1	0.200
WPMS10	F: GATGAGAAACAGTGAATAGTAAGA<br>R: GATTCCCAACAAGCCAAGATAAAA	$(GT)_{23}$	234–262	48°C	4	3	0.862	0.552	-0.56	0.001	3	0.902	0.599	-0.51	0.002

WPMS12	F: TTTTTCGTATTCTTATCTATCC	$(GT)_{19}$	157–183	48°C	4	4	0.241	0.743	0.675	0.286	2	0.033	0.379	0.914	0.264
	R: CACTACTCTGACAAAACCATC	$(GT)_{19}$	157–183												
WPMS14	F: CAGCCGCAGCCACTGAGAAATC	$(CGT)_{28}$	218-240	48°C	1	2	0.552	0.484	-0.14	0.004	3	0.673	0.642	-0.05	0.006
	R: GCCTGCTGAGAAGACTGCCTTGAC	$(CGT)_{28}$	218-240												
WPMS15	F: CAACAAACCATCAATGAAGAAGAC	$(CCT)_{14}$	180-206	48°C	1	3	0.454	0.503	0.097	0.011	3	0.627	0.587	-0.07	0.006
	R: AGAGGGTGTTGGGGGTGACTA	$(CCT)_{14}$	180-206												

A - number of observed alleles, H_O – observed proportion of heterozygotes, H_E - expected proportion of heterozygotes, F_{IS} - inbreeding coefficient ($F_{is} = 1 - H_O / H_E$), Null – null alleles

For markers ORPM30 and ORPM312 two different amplification sites were noted, the loci were designated in later analyses as ORPM30-1, ORPM30-2, ORPM312-1 and ORPM312-2.
