# Supplementary material for: Being Female Matters: Drivers of Spatial Genetic Structure in Clonal White Poplar
Source: Int J Mol Sci. 2026 Oct 9;27(20):8955. doi: 10.3390/ijms27208955 (PMC13704438; doi:10.3390/ijms27208955)
Supplement: Supplementary file 1 [file ijms-27-08955-s001.zip › Supplementary_file_2.pdf]

Among analysed SSRs loci we notice linkage disequilibrium between two pair of loci (ORPM21&ORPM312-2 and ORPM21&WPMS6) in both populations (Fig. S2). Verified in the poppr package.

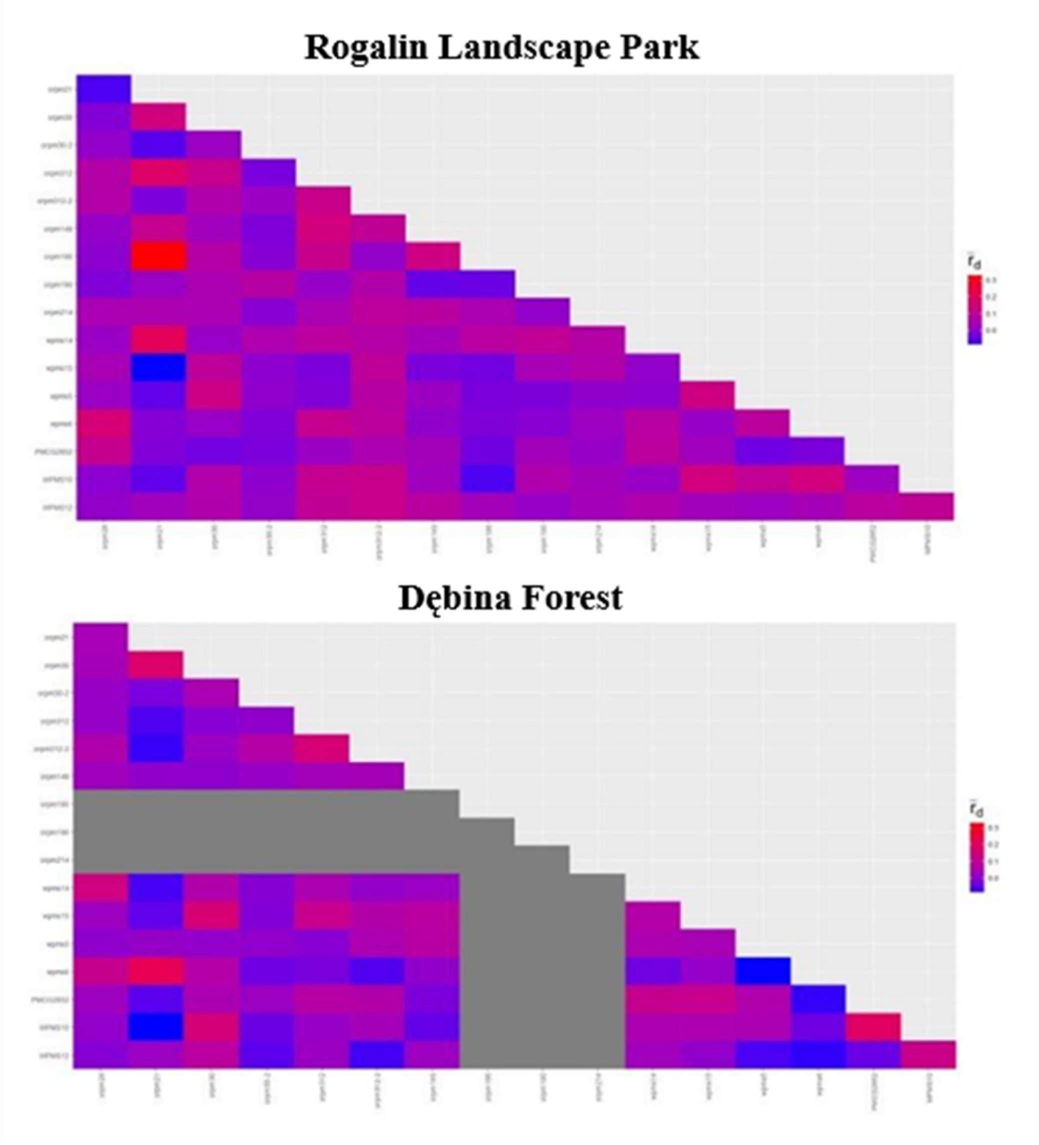

Fig. S1. Linkage disequilibrium - calculation of IA and r¯d for all pairs of loci.

Table S2. Two-sample Z-test results for differences in genetic diversity parameters (H_O, H_E, F_{IS}) between sexes and differences in clonal structure metrics between sexes and the whole population, in two study sites.

Parameter	Z-value	P-value	Significant	Comparsion
		Genetic diversity - Rogalin Landscape Park		

HO	22.37	0*	Yes	Male vs Female
HE	18.89	0*	Yes	Male vs Female
FIS	-5.66	1.47×10^{-8}*	Yes	Male vs Female
**Genetic diversity - Dębina Forest**				
HO	-13.10	0*	Yes	Male vs Female
HE	-13.18	0*	Yes	Male vs Female
FIS	-13.10	0*	Yes	Male vs Female
**Clonal structure – Rogalin Landscape Park**				
R	2.50	0.012	Yes	Female vs Male
D	0.46	0.643	No	Female vs Male
J	–12.86	0*	Yes	Female vs Male
**Clonal structure – Dębina Forest**				
R	–0.79	0.431	No	Female vs Male
D	–6.12	9.40×10^{-10}*	Yes	Female vs Male
J	–14.63	0*	Yes	Female vs Male

Z-value – Z statistic from two-sample Z-test, P-value – probability of observing the test statistic under the null hypothesis, H_O – observed proportion of heterozygotes, H_E - expected proportion of heterozygotes, F_{IS} - inbreeding coefficient ($F_{is} = 1 - H_O / H_E$), R - Clonal richness, D.- Simpson's diversity index, J - Pielou index, F – female, M - male; Significant results (p<0.05) are in bold; *** indicates p<0.001.

Table S3. Observed sex ratios and deviation from the expected 1:1 ratio, with chi-square test results.

Group	Female	Male	Ratio	Difference	Chi2	p-value	Expected
RLP (genets)	97	77	1.26	0.06	2.299	0.129	87:87
DF (genets)	74	79	0.94	-0.02	0.163	0.686	76.5:76.5
RLP (ramets)	270	231	1.17	0.04	3.036	0.081	250.5:250.5
DF (ramets)	279	288	0.97	-0.01	0.143	0.705	238.5:238.5

RLP – Rogalin Landscape Park, DF – Dębina Forest, Ratio – female-to-male ratio, Difference – deviation from equal sex ratio (female proportion – 0.5), Chi2 – chi-square statistic, p-value – significance level, Expected – expected female: male counts assuming 1:1 ratio.

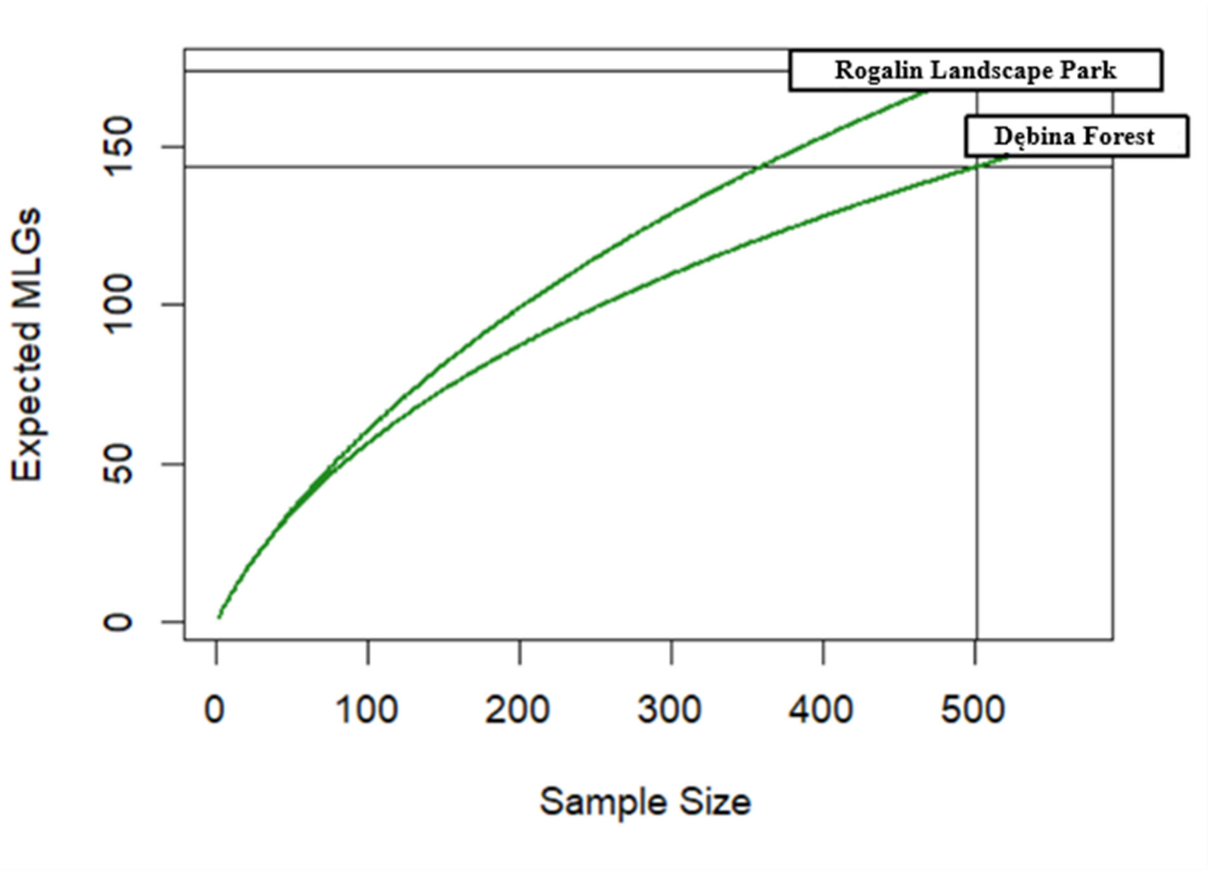

Fig. S2. Genotypic rarefaction curves showing the expected number of multilocus genotypes (eMLG) as a function of sample size for two populations: Rogalin Landscape Park and Dębina Forest. Curves were generated using rarefaction based on the smallest common sample size (minsamp) in the dataset.

For Rogalin Landscape Park, the Pareto index (β = 0.538) indicates a skewed clonal distribution, dominated by a few large genets and numerous small clones. A slightly higher Pareto index (β = 0.603) for Dębina Forest suggests a more even distribution of clone sizes compared to Rogalin Landscape Park, although a few large genets remain present.

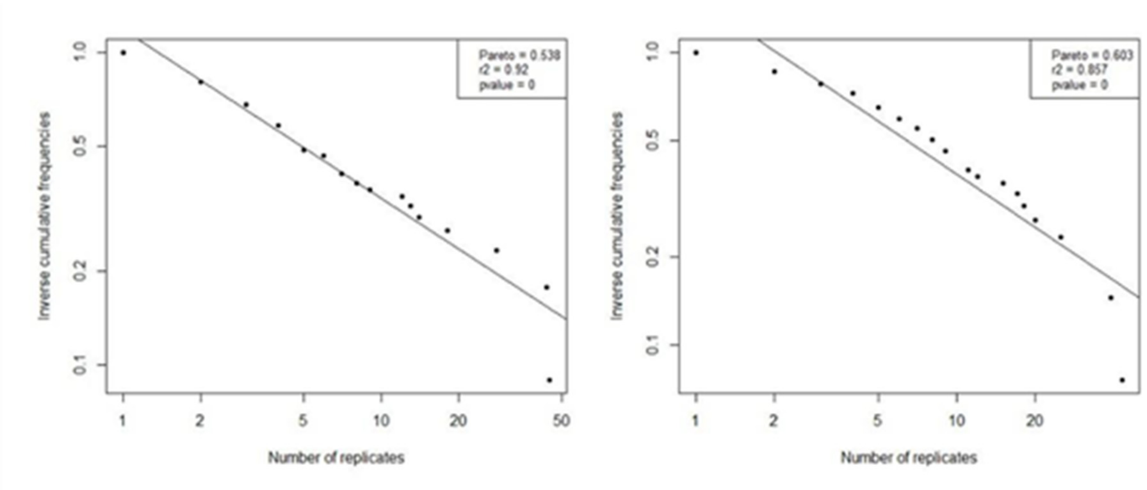

Fig. S3. The Pareto distribution for the Rogalin Landscape Park (left) and Dębina Forest (right).

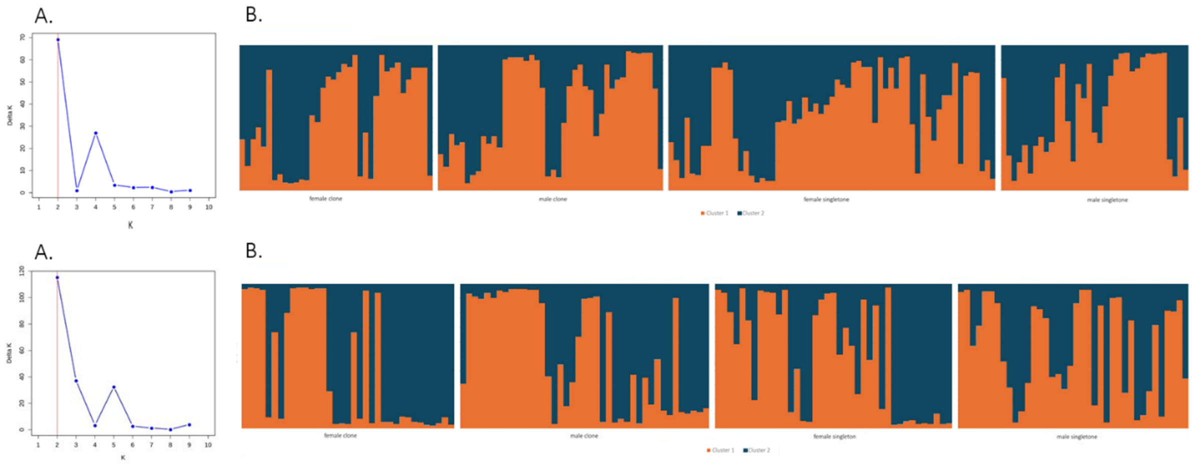

Fig.S4 (A) Most probable number of genetic groups (K), estimated using the ΔK method; (B) the proportion of each individual's membership in the detected genetic groups for Rogalin Landscape Park (upper panel) and Dębina Forest (lower panel) populations

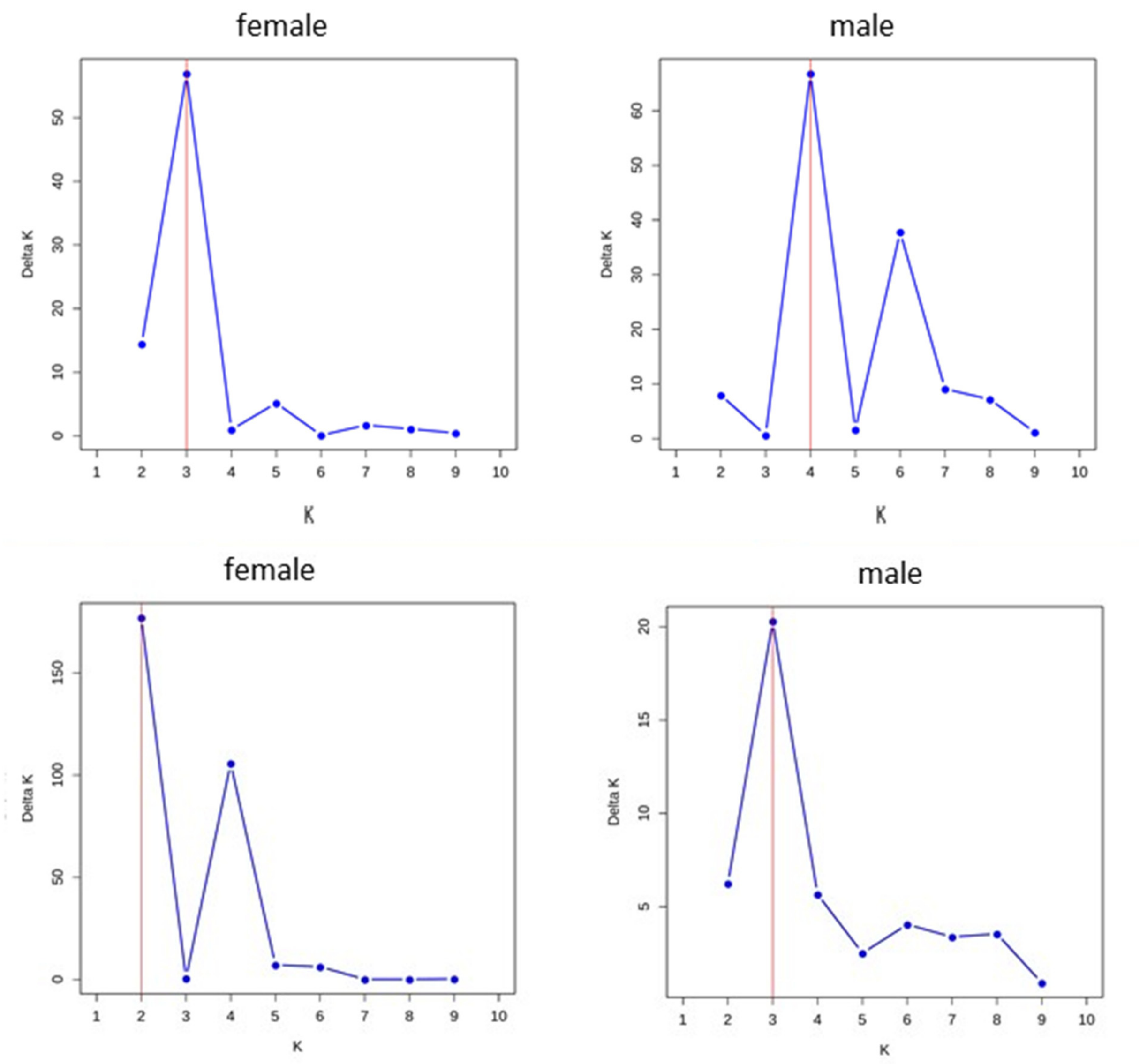

Fig. S5. Most probable number of genetic groups (K) for females and males individuals in the studied white poplar stands, estimated using the ΔK method, for Rogalin Landscape Park (upper panel) and Dębina Forests (lower panel) populations.

Table S4. Average cluster participation in two study sites: Rogalin Landscape Park (K = 4) and Dębina Forest (K= 3), based on STRUCTURE result for data not divided by sex.

Cluster participation	
**Rogalin Landscape Park**	
Cluster 1	0.38
Cluster 2	0.28

Cluster 3	0.18
Cluster 4	0.16
Total	1.00
**Dębina Forest**	
Cluster 1	0.30
Cluster 2	0.42
Cluster 3	0.28
Total	1.00

Table S5. Average distribution of sex and reproductive types within clusters in two study sites: Rogalin Landscape Park (K = 4) and Dębina Forest (K= 3), based on STRUCTURE result for data not divided by sex.

	Female clone	Female singletone	Male clone	Male singletone	Total
**Rogalin Landscape Park**					
Cluster 1	0.25	0.40	0.16	0.19	1.00
Cluster 2	0.24	0.36	0.21	0.19	1.00
Cluster 3	0.16	0.33	0.27	0.24	1.00
Cluster 4	0.10	0.23	0.47	0.20	1.00
**Dębina Forest**					
Cluster 1	0.23	0.20	0.33	0.24	1.00
Cluster 2	0.30	0.24	0.26	0.20	1.00
Cluster 3	0.13	0.33	0.21	0.33	1.00

Table S6. Average cluster participation in two study sites: Rogalin Landscape Park and Dębina Forest, based on STRUCTURE result for data divided by sex.

	Cluster participation	
**Rogalin Landscape Park**		
	female	male
Cluster 1	0.44	0.32
Cluster 2	0.22	0.27
Cluster 3	0.34	0.2
Cluster 4	-	0.21
Total	1	1
**Dębina Forest**		
	female	male
Cluster 1	0.54	0.42
Cluster 2	0.46	0.34
Cluster 3	-	0.24
Total	1	1

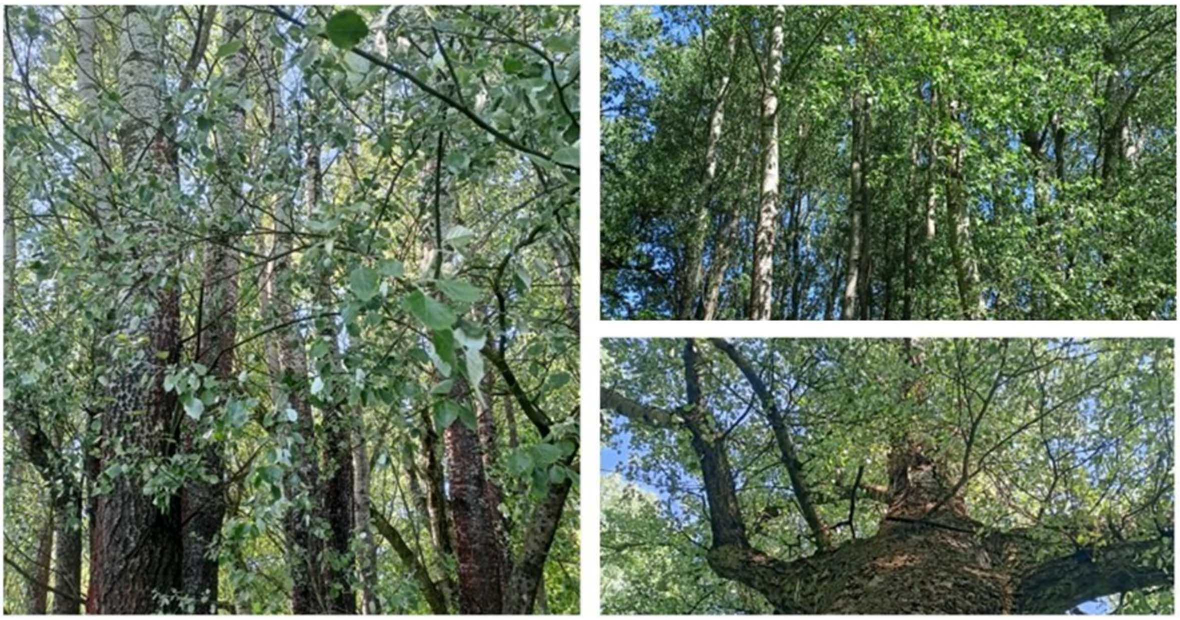

Fig. S6. Study site: Rogalin Landscape Park (Photo: MT)

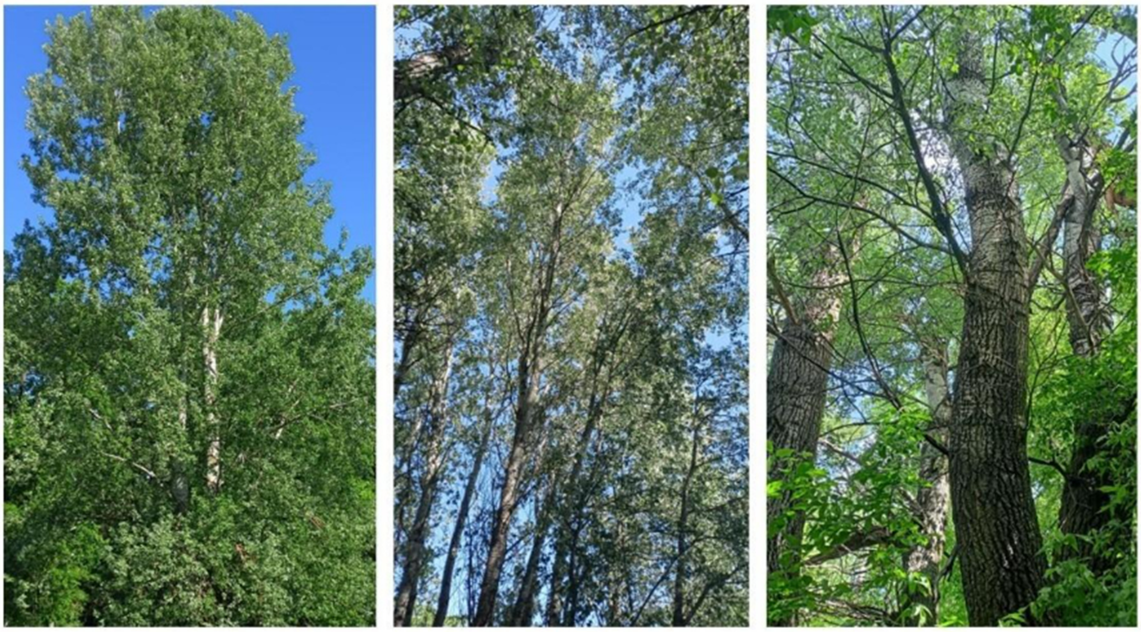

Fig. S7. Study site: Dębina Forest (Photo: MT)

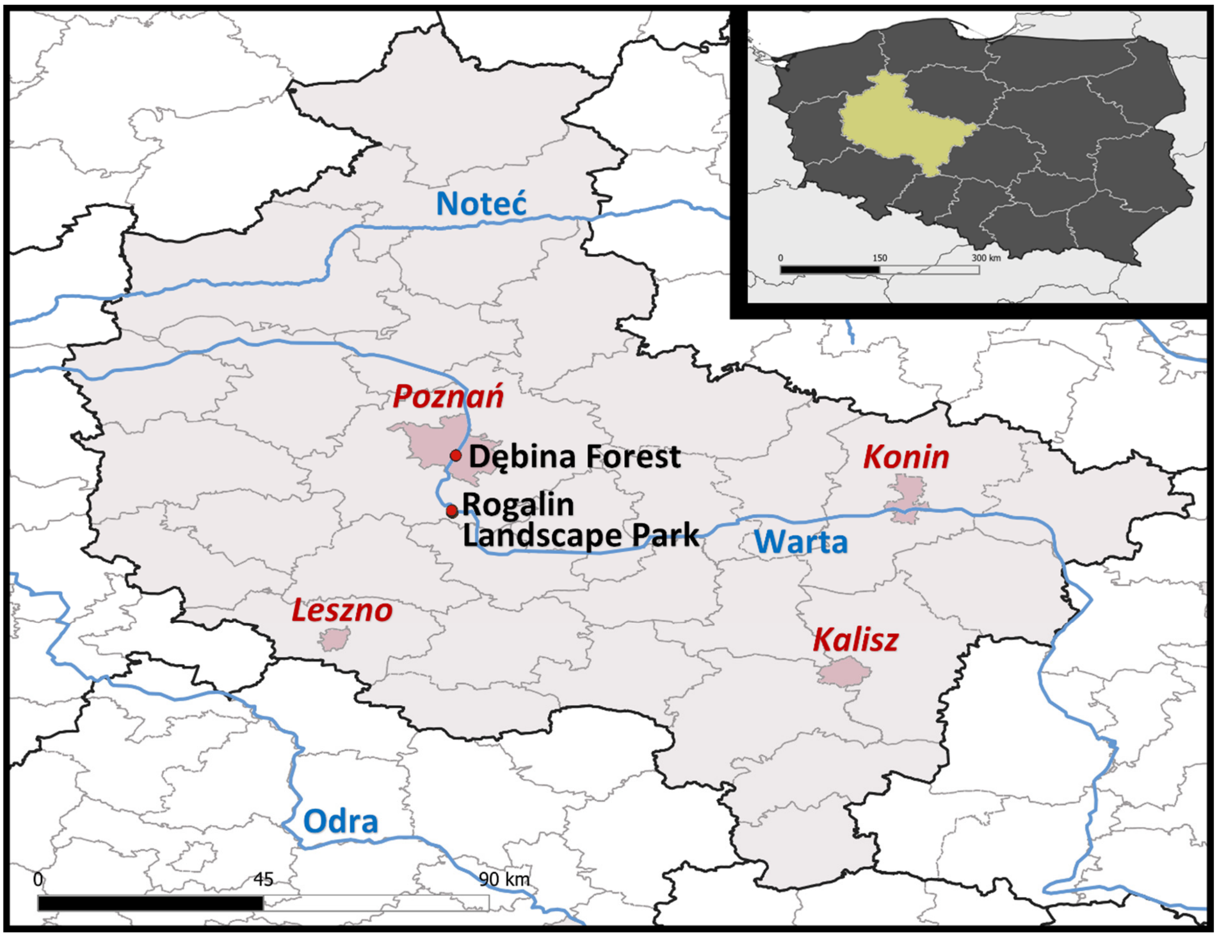

Fig. S8. General location of the white poplar study sites, Rogalin Landscape Park and Dębina Forest, in the Greater Poland Voivodeship. The top right inset shows the position of the region within Poland. Map generated in QGIS 3.44 (Qgis.org 2024).
